# How to design a complex behaviour change intervention: experiences from a nutrition-sensitive agriculture trial in rural India

**DOI:** 10.1136/bmjgh-2020-002384

**Published:** 2020-06-07

**Authors:** Helen Harris-Fry, Meghan O'Hearn, Ronali Pradhan, Sneha Krishnan, Nirmala Nair, Suchitra Rath, Shibanand Rath, Peggy Koniz-Booher, Heather Danton, Ashley Aakesson, Shibananth Pradhan, Naba Kishore Mishra, Abhinav Kumar, Avinash Upadhay, Audrey Prost, Suneetha Kadiyala

**Affiliations:** 1 Department of Population Health, London School of Hygiene and Tropical Medicine, London, UK; 2 Friedman School of Nutrition Science and Policy, Tufts University, Medford, Massachusetts, USA; 3 Digital Green, Bhubaneswar, India; 4 Ekjut, Chakradharpur, India; 5 John Snow Inc, Boston, Massachusetts, USA; 6 VARRAT, Keonjhar, India; 7 University College London Institute of Child Health, London, UK

**Keywords:** nutrition, child health, maternal health, Cluster randomised trial

## Abstract

Many public health interventions aim to promote healthful behaviours, with varying degrees of success. With a lack of existing empirical evidence on the optimal number or combination of behaviours to promote to achieve a given health outcome, a key challenge in intervention design lies in deciding what behaviours to prioritise, and how best to promote them. We describe how key behaviours were selected and promoted within a multisectoral nutrition-sensitive agriculture intervention that aimed to address maternal and child undernutrition in rural India. First, we formulated a Theory of Change, which outlined our hypothesised impact pathways. To do this, we used the following inputs: existing conceptual frameworks, published empirical evidence, a feasibility study, formative research and the intervention team’s local knowledge. Then, we selected specific behaviours to address within each impact pathway, based on our formative research, behaviour change models, local knowledge and community feedback. As the intervention progressed, we mapped each of the behaviours against our impact pathways and the transtheoretical model of behaviour change, to monitor the balance of behaviours across pathways and along stages of behaviour change. By collectively agreeing on definitions of complex concepts and hypothesised impact pathways, implementing partners were able to communicate clearly between each other and with intervention participants. Our intervention was iteratively informed by continuous review, by monitoring implementation against targets and by integrating community feedback. Impact and process evaluations will reveal whether these approaches are effective for improving maternal and child nutrition, and what the effects are on each hypothesised impact pathway.

**Summary box**

- Behaviour change interventions aiming to improve health outcomes can simultaneously focus on many, different behaviours.
- To decide on which behaviours to promote in our nutrition-sensitive agriculture intervention, we used the following inputs: existing conceptual frameworks and behaviour change models, published empirical evidence, a feasibility study, formative research, the team’s local knowledge and community feedback.
- As the intervention progressed, we mapped each of the prioritised behaviours against our hypothesised impact pathways and the transtheoretical model of behaviour change, to monitor the balance of behaviours across pathways and stages of behaviour change.
- Intervention design and implementation was aided by collective agreement between partners on definitions of complex concepts and hypothesised impact pathways, and the continuous review of implementation against targets and community feedback.

## Introduction

Many public health interventions aim to promote healthful behaviours, with varying degrees of success.^1 2^ Some interventions try to change behaviours by actively engaging participants using interactive techniques such as interpersonal counselling,^3^ motivational interviewing,^4^ or Participatory Learning and Action (PLA) with groups.^5–7^ Other approaches can be less interactive but reach a wider audience, for example, through mass media^8 9^ or text messaging.^10^

One challenge common to these interventions lies in deciding what behaviours to prioritise, and how best to change them, particularly when health outcomes are determined by several behaviours. For example, interventions aiming to reduce undernutrition could modify diets, physical activity, hygiene, or food purchasing and production decisions.^11^ Each of these could be divided into more specific behaviours, such as ‘eat one additional meal during pregnancy’ or ‘introduce complementary foods at six months of age’. It is easy to see how this could multiply, such that hundreds of behaviours could cumulatively improve a single health outcome. With increasing complexity of multisectoral interventions, such as nutrition-sensitive agricultural interventions, these options multiply even further and implementers have to make decisions regarding both agriculture and nutrition-specific impact pathways and behaviours. Implementers also have to decide how to adapt delivery platforms to address the multiple objectives of these complex interventions.

After selecting which behaviours to focus on, further analysis is required to identify how best to encourage their uptake. Enabling factors, which have been categorised by Michie, Van Stralen and West into people’s ‘capabilities’, ‘opportunities’ and ‘motivations’ (the ‘COM-B’ model),^12^ can vary in their relative importance across contexts, seasons and life stages. As articulated in the transtheoretical model, people may be at different stages in their process of adopting a behaviour—from thinking about it to trying it, and continuing with it.^13^

However, interventions cannot aim to change all relevant behaviours, or address all possible barriers. There are programmatic constraints on the number of activities an intervention can implement, and issues that an intervention can address, at an effective level of coverage. Moreover, addressing too many issues may be off-putting to participants—potentially causing information overload and choice fatigue, and inhibiting behaviour change.^14 15^

However, for health outcomes with a complex aetiology, we have limited understanding of how interventions should choose which behaviours to prioritise, or how many to promote. Tools such as the ‘Behaviour Change Wheel’ provide guidance on how to unpack the capabilities, opportunities and motivations that underlie a given behaviour.^12^ A few studies suggest that using more techniques to change behaviours increases intervention effectiveness,^1 16^ and we have some information on the relative effectiveness of varying doses and coverage. For example, increasing the number of participatory women’s groups per population increases impacts on neonatal mortality,^17^ and larger effects are also observed with increasing proportion of pregnant women attending groups.^18^ A recent evaluation of a radio programme promoting vitamin A-rich sweet potatoes found that around 44 episodes were needed to improve knowledge.^19^

Although increasing dosage, coverage and numbers of techniques may increase intervention effectiveness, there is little guidance on whether an intervention should aim to change many behaviours, or focus on just a few, or on how barriers to behaviour change should be prioritised and addressed.

## A case study from a nutrition-sensitive agriculture trial in India

In this paper, we provide a case study of a complex nutrition-sensitive agriculture (NSA) intervention that aimed to reduce maternal and child undernutrition in rural India. Specifically, we describe how we (1) identified the six most important pathways through which we hypothesised our interventions would improve nutrition outcomes, (2) prioritised behaviours and barriers to behaviour change to address within these pathways, (3) reviewed our intervention content against these priorities and (4) continually integrated community participants’ reported priorities.

The Upscaling Participatory Videos and Action for Agriculture and Nutrition (UPAVAN) trial is a four-arm cluster-randomised controlled trial aiming to improve maternal and child nutrition through the dissemination of locally developed videos, women’s group meetings and follow-up home visits in rural Odisha, India. The primary outcomes are % children aged 6–23 months consuming at least four food groups per day and maternal body mass index.

UPAVAN has seven partners. Digital Green coordinated intervention implementation; Voluntary Association for Rural Reconstruction and Appropriate Technology (VARRAT) implemented the interventions; JSI Research and Training Institute provided technical assistance on behaviour change, formative research and training; and Ekjut provided technical assistance on a PLA component. London School of Hygiene & Tropical Medicine led all research activities, in collaboration with University College London and Development Corner Consulting.

Full details of the trial design are given in the protocol^20^ and an operational protocol detailing roles and responsibilities, intervention content, coverage, dosage, timings, monitoring systems and training plans is available in the online supplementary file 1.

10.1136/bmjgh-2020-002384.supp1 Supplementary data

### Study context, rationale and overview

Rates of undernutrition in India are high: 38% of children under 5 years of age are chronically undernourished (height-for-age z-score <−2 SD), a fifth are acutely undernourished (weight-for-height z-score <−2 SD), and around a quarter of women are underweight (body mass index <18.5 kg/m^2^).^21^

Agriculture can play a role in improving nutrition outcomes. Programmes promoting home production of nutrient-rich foods (biofortification, homestead gardens, livestock rearing) have increased dietary diversity.^22 23^ Inclusion of behaviour change and women’s empowerment interventions, in particular, has been key to enhancing the impact of agriculture on nutrition outcomes.^22–24^

**Figure 1:**
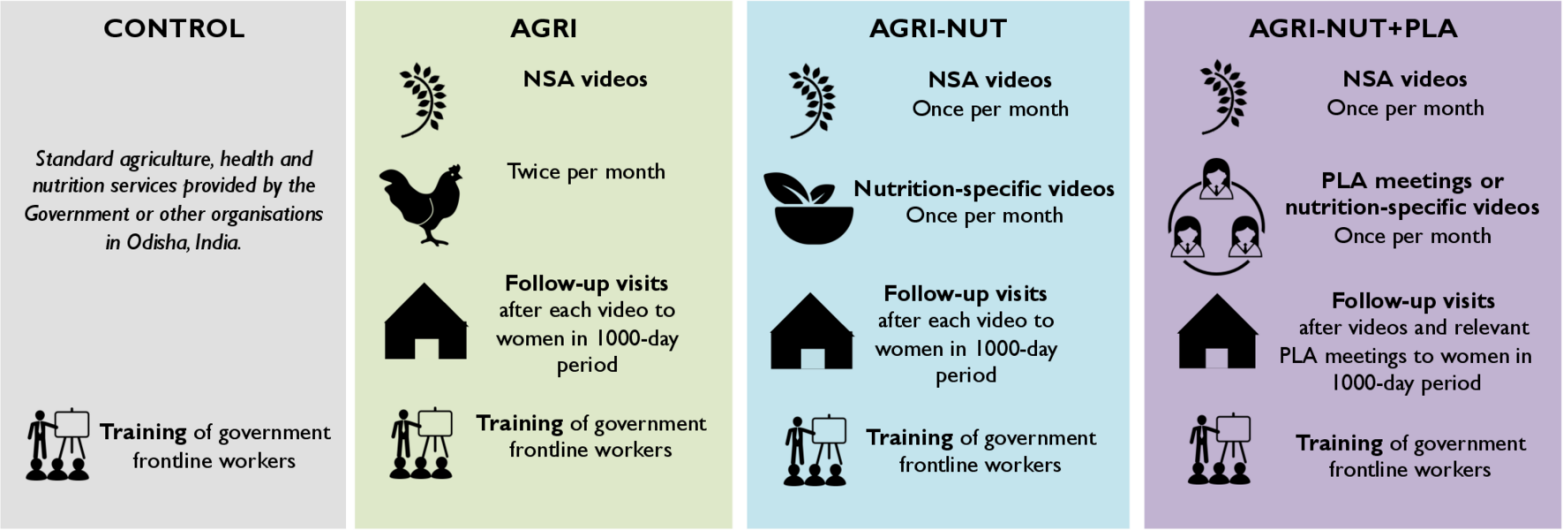
Overview of Upscaling Participatory Videos and Action for Agriculture and Nutrition (UPAVAN) interventions, taken from Kadiyala *et al*.^20^ NSA, nutrition-sensitive agriculture; PLA, Participatory Learning and Action.

Digital Green—a global development organisation—has developed an agricultural intervention involving community-led production and dissemination of videos in community groups. This has resulted in large improvements in agricultural practices and agricultural productivity in Bihar, India.^25^ During an earlier study by the Strengthening Partnerships, Results and Innovations Globally (SPRING) project, we found it was feasible to integrate content on maternal and child nutrition into similar agricultural videos.^26^ UPAVAN tests whether three ‘nutrition-sensitive’ agriculture (NSA) variants of this approach (figure 1) can improve maternal and child nutrition in Odisha, India.

The three intervention variants are:

1. Fortnightly women’s groups viewing and discussing videos on NSA practices, and home visits to encourage the adoption of new practices shown in videos.
2. Fortnightly women’s groups viewing and discussing videos on NSA, and ‘nutrition-specific’ behaviours (without agriculture content), plus home visits.
3. Fortnightly women’s groups viewing and discussing videos on NSA and nutrition-specific behaviours, combined with a cycle of PLA group meetings, plus home visits. With help from the videos, these PLA meetings encouraged members to collectively understand the problem of undernutrition, and then identify, prioritise and act on locally feasible solutions to address this problem.

Local VARRAT staff facilitated the video disseminations, PLA meetings and home visits, and the interventions were open to all women in the community, although men were not discouraged from observing the video disseminations and PLA meetings. All interventions began with community mobilisation activities, and training of programme staff on maternal and child nutrition, hygiene and NSA.

Each intervention is compared with a control arm receiving standard government services and a 2-day nutrition training to government female community health workers, provided in all arms. The interventions began in April 2017 and ended in November 2019.

### Unpacking possible behaviours to prioritise

**Figure 2:**
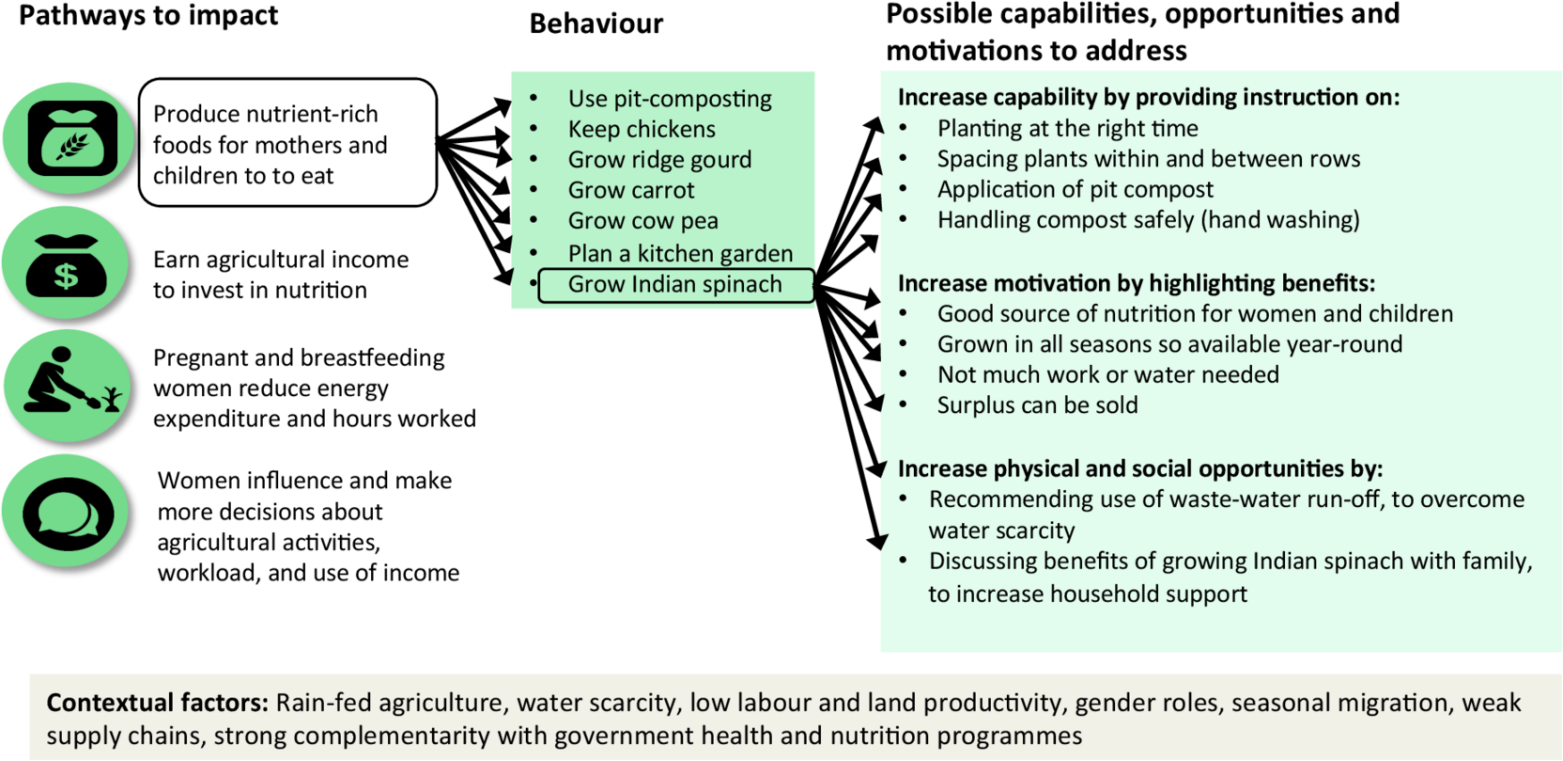
Unpacking the possible pathways, behaviours and capabilities, opportunities and motivations that Upscaling Participatory Videos and Action for Agriculture and Nutrition (UPAVAN) could prioritise.

The UPAVAN trial provides an apt example of the multiple pathways, behaviours and associated capabilities, opportunities and motivations that could change a single health outcome, as illustrated in figure 2.

There are several ways that NSA could improve nutrition outcomes (left-hand box; figure 2). Within a pathway there are many crops or agricultural behaviours that we could promote (middle box; figure 2), and people may have various capabilities, opportunities or motivations that encourage or improve behaviour change (right-hand box; figure 2).

### Stages of intervention development

**Figure 3:**
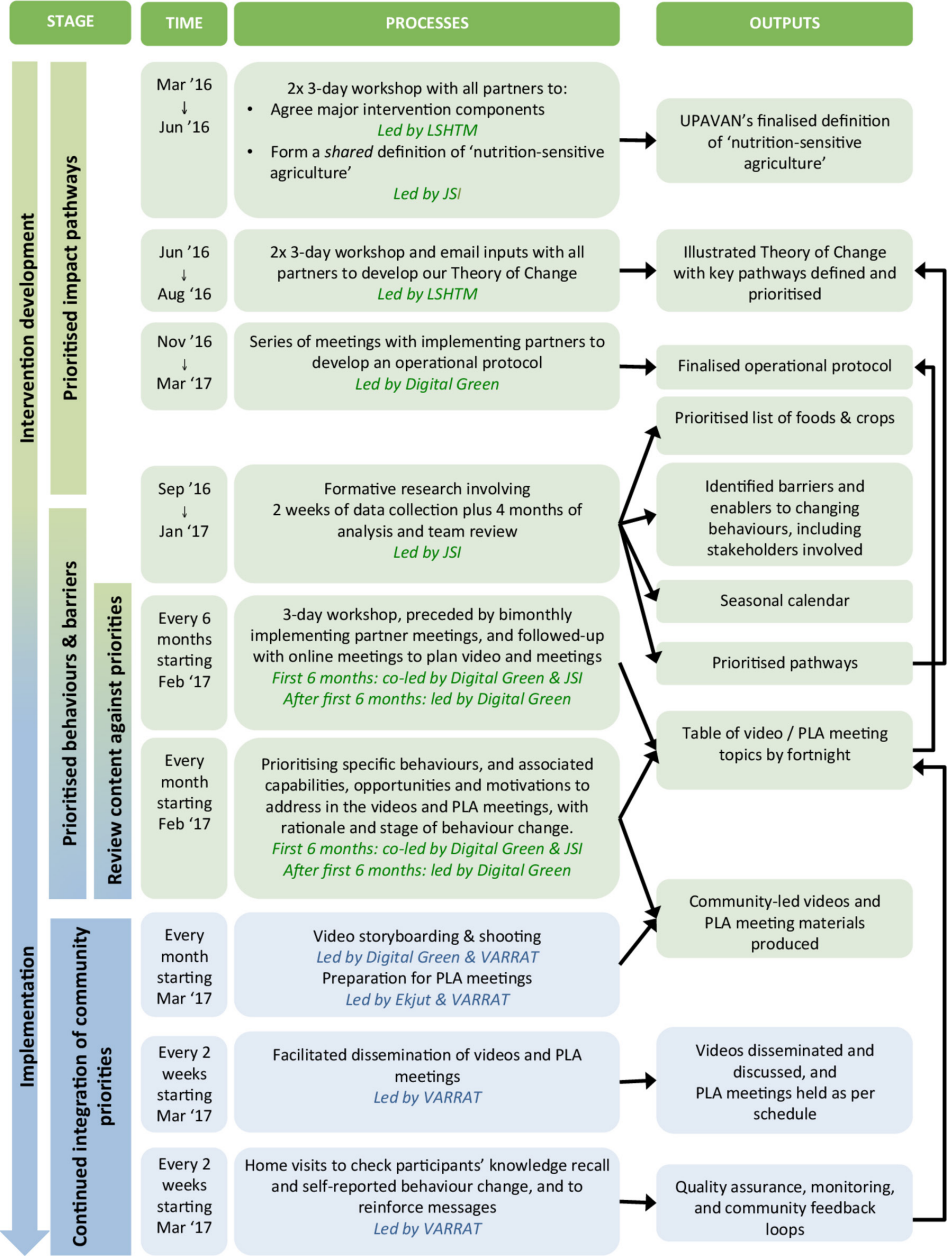
Key stages in the development and prioritisation of Upscaling Participatory Videos and Action for Agriculture and Nutrition (UPAVAN) interventions. PLA, Participatory Learning and Action; VARRAT, Voluntary Association for Rural Reconstruction and Appropriate Technology.

Figure 3 shows the key stages by which we prioritised which topics to address in UPAVAN’s videos, PLA meetings and home visits.

First, we hypothesised which NSA and nutrition-specific pathways were most likely to improve our outcomes by collectively developing a definition of ‘nutrition-sensitive agriculture’, and a Theory of Change that outlined possible impact pathways. Next, we prioritised specific behaviours within these pathways. This was informed by published evidence and formative research.^27^ An operational protocol of implementation processes kept the team focused on the prioritised pathways and behaviours. Finally, from the prioritised pathways (in the Theory of Change) and associated prioritised NSA and nutrition-specific behaviours (from the formative research), we identified capabilities, opportunities and motivations to be addressed in each video or PLA meeting and associated home visit.

## Prioritising impact pathways

To decide on our priority pathways, we used the following inputs: conceptual frameworks, published empirical evidence, a feasibility study, formative research and local knowledge from VARRAT, Digital Green and Ekjut team members.

### Existing conceptual frameworks

Several conceptual frameworks on the links between agriculture and nutrition already exist.^28–30^ We grounded our working definition of ‘nutrition-sensitive agriculture’ and our Theory of Change in these frameworks, and only included pathways and practices relevant for our study. Using Kadiyala *et al,*^28^ we ignored macro-level factors such as domestic food trade, health infrastructure and employment, which would not realistically be influenced by our intervention. Instead, we focused on household and intra-household-level factors, such as income, agricultural production, food expenditures, caring capacity and practices, and women’s empowerment and energy expenditure.

### Existing empirical evidence

In particular, we drew from nutrition interventions in India,^5^ Nepal^7 31^ and Bangladesh,^9^ and homestead gardening interventions.^32 33^ Using existing evidence, we deprioritised two possible pathways on hygiene and care-seeking during illness. Although infection intuitively seems like an important factor to address and is an important determinant of nutritional status,^34 35^ a PLA group intervention in India improved hand washing practices but had no effect on child illness,^5^ and another trial found no effects of a water, sanitation and hygiene intervention on child length,^36^ perhaps due to multiple exposures to infection risks. Therefore, we chose not to focus on infection reduction, but did emphasise the importance of hand washing as a preventative measure, in relation to promoted agricultural practices, such as compost making and chicken farming.^37^ The PLA intervention also found that seeking care from a nurse or doctor was not amenable to change,^5^ and we were constrained by supply-side limitations in the quality of care, so we also deprioritised this pathway.

### Feasibility study and formative research

We conducted a feasibility study^38^ and formative research^27 39^ before starting intervention activities. The formative research was led by technical experts, JSI Research and Training Institute, in collaboration with implementing teams (VARRAT, Digital Green and Ekjut), and aimed to (1) understand community members’ perspectives; (2) develop a list of foods, crops and livestock; (3) identify barriers and enablers to changing agriculture and nutrition behaviours, including stakeholders involved; and (4) create a seasonal calendar of agricultural processes, cash flows, labour and gender roles. Methods used were focus group discussions, a participatory food ranking using pile sorts, an exercise to fill out daily activity charts for participants and their family members, and direct observation via transect walks through selected villages.

This provided contextual information on which pathways might be relevant and amenable to change. For example, daily activity charts created by women, their husbands and mothers-in-law showed that a major issue was the heavy work burdens that women of reproductive age faced, compared with men and older women. Focus group discussions indicated that it could be feasible to reduce women’s workload, and heavy or time-consuming tasks could be shifted to other adults in the household.

### Local knowledge from the implementation team

We also drew from the local team’s knowledge about agricultural and nutrition practices and barriers to change. For example, we did not prioritise improving value-chain pathways (eg, improving cold storage facilities for agriculture produce), because the team considered it unfeasible to change in the time frame. Instead, we focused on increasing agricultural income, since agriculture is the main livelihood,^40^ and income is an important constraint to agricultural productivity and dietary adequacy.

This left us with four prioritised NSA pathways (in figure 2) and two nutrition-specific pathways (improving maternal diets and improving child feeding practices). This Theory of Change, and methods of measuring these pathways, are given in our protocol.^20^

## Prioritising behaviours and barriers

After prioritising our key pathways, we needed a second round of prioritisation to select specific behaviours to address. We initially drew on our formative research, local knowledge and impact pathways.

### Formative research and local knowledge

To prioritise the NSA behaviours, we overlaid the seasonal calendar with the crop and food list, and prioritised foods and crops based on:

- Time of year.
- Nutritive value.
- Economic value.
- Labour requirements.
- Cost and accessibility of inputs required.
- Feasibility of adoption.

For example, based on the seasonal calendar and local agronomic feasibility, videos on cultivating Indian spinach with wastewater and locally available seeds were disseminated during lean seasons when water is scarce.

To identify which nutrition-sensitive agricultural behaviours to promote within a prioritised food or crop, we identified what single, feasible change was most important. Similar to Berti *et al,*^41^ the local team determined whether the food or crop was new to the area but could be promoted (such as carrots), or whether it was already produced but practices could be improved (eg, improving spacing of Indian spinach, or encouraging people to eat pumpkins that they already grow).

We then filled gaps in the video and PLA meeting calendar with behaviours that were less time-sensitive but nevertheless important, such as videos explaining the concept of NSA, or household budgeting.

For each behaviour, we identified reasons why people were not already doing the practice. For example, our formative research found a belief in some communities that Indian spinach is harmful for children and pregnant women, often imposed by mothers-in-law. The videos therefore featured a story of a mothers-in-law’s journey of ‘pre-contemplation’ to ‘action’, during which she became an advocate for the consumption of Indian spinach.

The team also identified ways to address multiple pathways concurrently. For example, a series of videos focused on producing crops that require minimal labour and are of economically high value, such as mushrooms. Growing mushrooms could supplement income and reduce women’s energy expenditure.

Recognising that the prevalence, appropriateness, and feasibility of nutrition and agricultural practices would vary by multiple factors (such as geography, caste, wealth and season), we tried to capture a breadth of perspectives in the formative research. When facilitating the group discussions, meeting content was tailored to be specific to the participants. Group members discussed barriers and solutions to adoption that may be more or less relevant for them (eg, water scarcity, restrictive gender roles) and shared their experiences with each other. The PLA meetings are specifically designed to be locally appropriate because groups themselves identify and prioritise salient problems, solutions and strategies to implement.

### Reviewing intervention content in relation to our priorities

We reviewed the video content and group meeting plans every 6 months, and assessed whether we were giving each pathway equal weight. We found some pathways easier to address than others. Notably, the promotion of nutrient-rich foods for household consumption was conceptually simple, whereas increasing women’s decision-making power in the household proved difficult to conceptualise, storyboard and film.

We also mapped each specific promoted behaviour against the transtheoretical model of behaviour change,^13^ based on whether the behaviour was generally (1) new to the community (‘pre-contemplation’); (2) being considered (‘contemplation’); (3) of interest to the community (‘preparation’), (4) being first adopted (‘action’); (5) being continued (‘maintenance’); or (6) being modelled to others in the community (‘termination’).

Table 1 illustrates this mapping using a series of videos on chicken farming.

**Table 1:** An example of mapping videos, with specific behaviours, capabilities, opportunities and motivations addressed, to the main pathway and transtheoretical behaviour change stage

Title of the video	Main prioritised pathway	Specific behaviours promoted	Capabilities, opportunities and motivations addressed	Transtheoretical behaviour change stage
Benefits of chicken farming	Produce food	Raise chickens Pregnant women and children eat the meat and eggs produced	**Motivate** participants to raise chickens by highlighting benefits (source of income and nutritious food)	Contemplation
How to practise improved chicken farming	Earn income	Keep chickens in a small house, especially at night, to keep them safe and so they lay more eggs Families decide together who should care for the chickens and what to do with the produce and income from surplus	Increase **capabilities** to improve chicken farming by providing instruction on penning at night Increase women’s social **opportunities** to be involved in decisions about workload and use of chicken produce and income by promoting joint decision-making	Preparation
Benefits of chicken farming—Testimonial	Produce food	Regularly immunise chickens to ensure high survival rates Pregnant and breastfeeding women, and children aged 6–24 months should consume eggs at least every other day Sell surplus chicks, chickens and eggs only after there is enough for women and children to consume eggs at least every other day	**Motivate** participants to keep chickens and immunise them by sharing a success story Increase **capability** to keep healthy chickens and improve diets by providing instruction on immunisation and egg consumption	Action
How to practise improved chicken farming	Produce food	Keep chickens in a small house, especially at night, to keep them safe and so they lay more eggs Families should decide together who should care for the chickens and what to do with the produce	Increase **capability** to improve chicken farming by reinforcing instruction on penning Increase women’s **opportunities** to be involved in decisions about workload and use of chicken produce	Maintenance

This mapping exercise allowed us to be systematic in ensuring balance across pathways and track behaviour change stages, and kept us focused on a confined, core set of behaviours.

### Continued integration of community priorities

**Figure 4:**
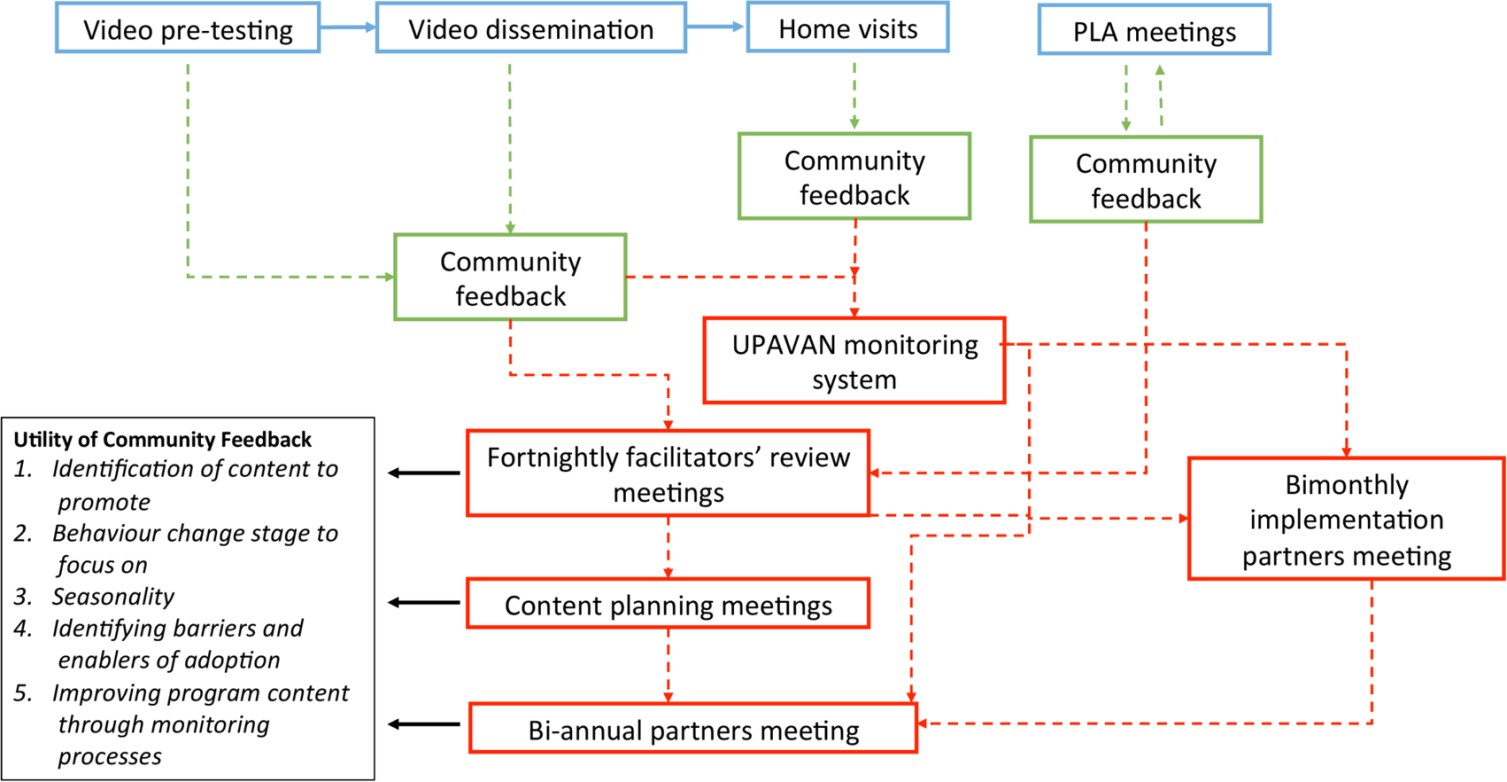
Flow of community feedback in Upscaling Participatory Videos and Action for Agriculture and Nutrition (UPAVAN). PLA, Participatory Learning and Action.

Once the intervention was underway, we drew heavily on community feedback (figure 4). To balance expert opinion, evidence and community priorities, we often arrived at decisions through consultation with the group meeting facilitators.

To create the videos, we developed a storyboard based on our agreed behaviours and barriers, written in local Odia language, and then filmed with community members. We collected feedback from facilitators, local government health workers (Anganwadi workers and agricultural extension workers) and protagonists. After showing the videos, facilitators discussed with participants the appropriateness of the videos, reasons why they may not adopt promoted behaviours and ideas of new topics, all of which informed future videos.

Unique to the PLA meetings, an additional level of prioritisation occurred in each women’s group. We used a list of nutrition-specific behaviours identified from the formative work and local knowledge to create picture cards for participants to collectively discuss, prioritise and find strategies to implement. This meant that, in this intervention component, PLA groups would discuss a common set of topics related to maternal and child undernutrition, but each group differently prioritised which problems they wanted to address and strategies to implement.

Unlike in the group meetings where women may feel shy to speak up, home visits gave an opportunity for participants to quietly discuss the relevance of, and enablers and barriers to, promoted behaviours. During the home visits, facilitators also collected data on participants’ recall of the messages shown in videos or discussed in meetings, and their adoption of promoted behaviours.

This feedback was collated by the VARRAT team during fortnightly review meetings with the group facilitators, and used to plan content and review progress. The quantitative data collected at the home visits were analysed by Digital Green to quantify coverage gaps, knowledge recall and behaviour adoption.

Based on feedback from participants and group facilitators, new topics were introduced and popular topics were repeated. For instance, we did not plan to promote behaviours on limiting unhealthy snacks, but qualitative feedback identified unhealthy snacks displacing nutrient-rich foods as a concern. Therefore, we included this as a new topic in videos and PLA meetings. A popular topic that we repeated was Indian spinach. Monitoring data from the home visits found that it was popular: 1344 (11.5% of video viewers) households adopted it after the first video, and qualitative feedback also showed community demand for another video. Therefore, a second video was developed to reinforce the benefits of growing Indian spinach with improved cultivation practices. Monitoring data showed that adoption more than doubled to 2932 (15.2%) households.

## Reflections on lessons learnt

We reflect on four key lessons learnt from our experience of prioritising behaviours and barriers to address in a complex behaviour change intervention.

First, our shared understanding of complex concepts such as ‘nutrition-sensitive agriculture’, ‘women’s empowerment’ and pathways to impact across all partners enabled group facilitators to understand and clearly communicate these concepts. The consensus-based approach in developing the Theory of Change enabled mutual knowledge exchange between partners and was instrumental in subsequently structuring the video disseminations and PLA meeting plans. Our Theory of Change was the guiding framework for designing, prioritising and communicating complex behaviours.

Second, there is a delicate trade-off between time taken to design the intervention and time taken to reach consensus across partners. We spent around 1 year on the set-up of this intervention, which included formative research. Time-consuming factors were the complexity and number interventions (as this was a four-arm trial), our consensus-based approach and, relatedly, UPAVAN’s large number of partner organisations. However, we think this investment in set-up resulted in efficiencies later on, due to less reliance on international technical expert inputs, smooth intervention delivery and equitable partnerships. There were also factors that ensured we used this set-up time efficiently. Our interventions built on pre-existing models of implementation (Digital Green’s video approach and PLA), we had already conducted a feasibility study, and the implementing partners had extensive local knowledge.

Third, there is a related balance between using inputs from technical experts while staying true to the community-led, participatory principles inbuilt in Digital Green’s video-making and in PLA. In UPAVAN, key decisions about intervention design that we discussed at length, but we feel were worth the time, regarded the intervention dosage (number of meetings per month and number of groups per population) and prioritising which pathways to focus on. Since complex programmes often involve programmatic trade-offs, and ideological or epistemological differences among programme partners, reaching consensus was important and empowering to all partners involved.

Finally, for such complex interventions to work, fidelity to the implementation design is essential. Continuous review with a strong monitoring system—that integrated community feedback, mapped the Theory of Change and behaviour change stages, and assessed progress against agreed targets—was both essential and doable. With time, local implementers were able to lead this review in a self-reflective process.

## Conclusion

This article documented the process through which the team leading a complex NSA trial (UPAVAN) selected key pathways to impact, and identified which behaviours to promote and barriers to address. Key inputs to these processes were existing conceptual frameworks and behaviour change models, empirical evidence, feasibility and formative research, a collectively agreed on Theory of Change, local knowledge of implementing partners, and community feedback. We found the most useful inputs were the shared understanding of impact pathways and strong community feedback loops.

Impact and process evaluations will reveal whether these approaches to prioritise behaviours and design a behaviour change intervention are effective for improving maternal and child nutrition, and the pathways by which the interventions did or did not work.
