# Supplementary material for: How to design a complex behaviour change intervention: experiences from a nutrition-sensitive agriculture trial in rural India
Source: BMJ Glob Health. 2020 Jun 7;5(6):e002384. doi: 10.1136/bmjgh-2020-002384 (PMC7282327; doi:10.1136/bmjgh-2020-002384)
Supplement: Supplementary data [file bmjgh-2020-002384supp001.pdf]

UPAVAN Operational Protocol

Table of Contents

1. Trial overview 2
2. Intervention development and the process of topic prioritization 5
3. Video dissemination 6
4. Participatory Learning and Action (PLA) in Arm 3 8
5. Home visits 11
6. Frontline worker training 12
7. Women's group formation: community engagement, and mobilization of target beneficiaries 12
8. Process of PoP and video development 14
10. Intervention delivery time plan 17
11. Operational steps for trial Integrity 20

Appendices

Appendix 1: UPAVAN operational definitions and acronyms

Appendix 2: UPAVAN Monitoring Information System and protocol to ensure quality and adherence to implementation protocol

Appendix 3: UPAVAN operational plan for training

Harris-Fry H, *et al. BMJ Global Health* 2020; 5:e002384. doi: 10.1136/bmjgh-2020-002384

1. Trial overview

UPAVAN will be conducted in in 148 clusters in four blocks of Keonjhar district (Patna, Keonjhar, Harichandanpur, and Ghatagaon blocks). The blocks are shown in Figure 1. The clusters were created by DG and VARRAT, merging some villages to give approximately equal cluster sizes, and LSHTM have randomly allocated them to each of the four trial arms (37 clusters per arm).

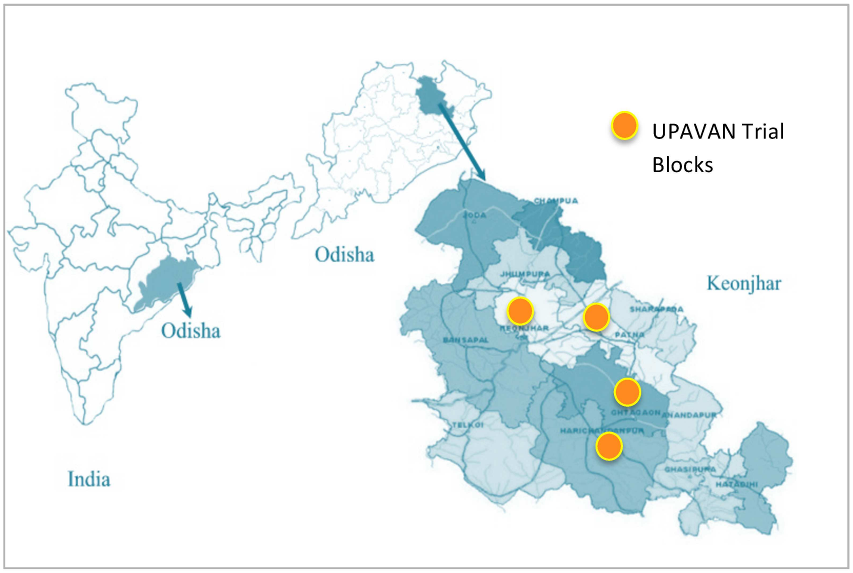

Figure 1 Map of UPAVAN study site

The trial arms are illustrated in Figure 2:

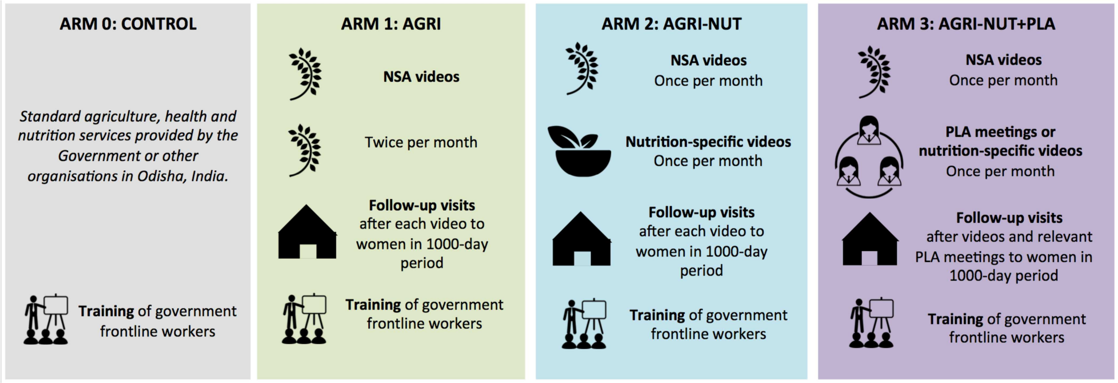

Figure 2 Overview of intervention arms

Harris-Fry H, *et al. BMJ Global Health* 2020; 5:e002384. doi: 10.1136/bmjgh-2020-002384

Arm 0: Control

- Standard services that are provided by the Government or other organizations, delivered as usual.
- Basic training to government frontline workers (ASHAs and AWWs) on maternal, infant and young child nutrition

Arm 1: AGRI

An agricultural extension intervention of:

- Fortnightly* low-cost videos on nutrition-sensitive agriculture (NSA), disseminated in women's groups (20-25 members per group) with facilitated discussions
- Fortnightly* home visits to all women in the 1000-day period to reinforce messages and encourage uptake of new practices shown in the videos
- Basic training to government frontline workers (ASHAs and AWWs) on maternal, infant and young child nutrition

Arm 2: AGRI-NUT

An agricultural extension intervention of:

- Monthly* low-cost videos on nutrition-sensitive agriculture PLUS monthly* videos on nutrition-specific practices, disseminated in women's groups (20-25 members per group) with facilitated discussions
- Fortnightly* home visits to all women in the 1000-day period to reinforce messages and encourage uptake of new practices shown in the videos
- Basic training to government frontline workers (ASHAs and AWWs) on maternal, infant and young child nutrition

Arm 3: AGRI-NUT+PLA

An agricultural extension intervention of:

- Monthly* low-cost videos on nutrition-sensitive agriculture, disseminated in women's groups (20-25 members per group) with facilitated discussions, PLUS
- Monthly* women's group meetings following a 'Participatory Learning and Action' (PLA) cycle, with discussion-based meetings in larger PLA groups (35-40 members) or viewing and facilitated discussion of PLA-generated nutrition-specific videos in the smaller video viewing groups (20-25 members per group, i.e., those that view the NSA videos).
- Fortnightly home visits after videos that are relevant for home visits (all NSA videos and phase III of PLA cycle)
- Basic training to government frontline workers (ASHAs and AWWs) on maternal, infant and young child nutrition

** Implementation will have breaks in implementation, based on seasonal factors, festivals, or other contextual factors. We anticipate 20 out of 24 fortnightly disseminations will be delivered per year.*

Harris-Fry H, *et al. BMJ Global Health* 2020; 5:e002384. doi: 10.1136/bmjgh-2020-002384

All intervention arms receive:

- **Fortnightly group meetings, of varying content.**
- **A minimum of monthly nutrition-sensitive agriculture videos** on a pre-defined set of prioritised practices.
- **Home visits to all women in the 1000-day period** to reinforce messages, encourage uptake of new behaviours, promote group attendance, and monitor intervention progress.
- **Basic training to government frontline workers** on maternal, infant and young child nutrition

The prioritized UPAVAN nutrition-sensitive and nutrition-specific topics are summarised in Table 1:

Table 1 UPAVAN's prioritised nutrition-sensitive and nutrition-specific practices

Nutrition-sensitive agriculture topics	Nutrition-specific topics
– Increase production and diversity of seasonal and prioritised nutrient-rich crops and livestock<br>– Enhance household-level income from agricultural activities<br>– Women and their family members make joint decisions about agricultural activities, workload and using income for health and nutrition<br>– Labour sharing to reduce workload of pregnant and lactating women<br>– Process and store key crops to ensure their safety, stop nutrient loss, and extend availability<br>– Safely process animal faeces to make organic manure and improve sanitation<br>– Safely use pesticides and fertilisers<br>– Women and their family members make joint decisions about<br>– Use labour saving agricultural practices to reduce maternal workload	– Pregnant women and women with children under 2 years improve the quantity and quality of their diets<br>– Rest during pregnancy and post-partum<br>– Breastfeed exclusively until 6 months of age<br>– Caregivers introduce complementary foods at 6 months<br>– Feed children age-appropriate complementary foods<br>– Feed children appropriately during and after illness, including ORS if needed

Harris-Fry H, *et al. BMJ Global Health* 2020; 5:e002384. doi: 10.1136/bmjgh-2020-002384

2. Intervention development and the process of topic prioritization

Figure 1 describes the process by which the UPAVAN team will prioritize NSA and nutrition-specific practices for videos and PLA content:

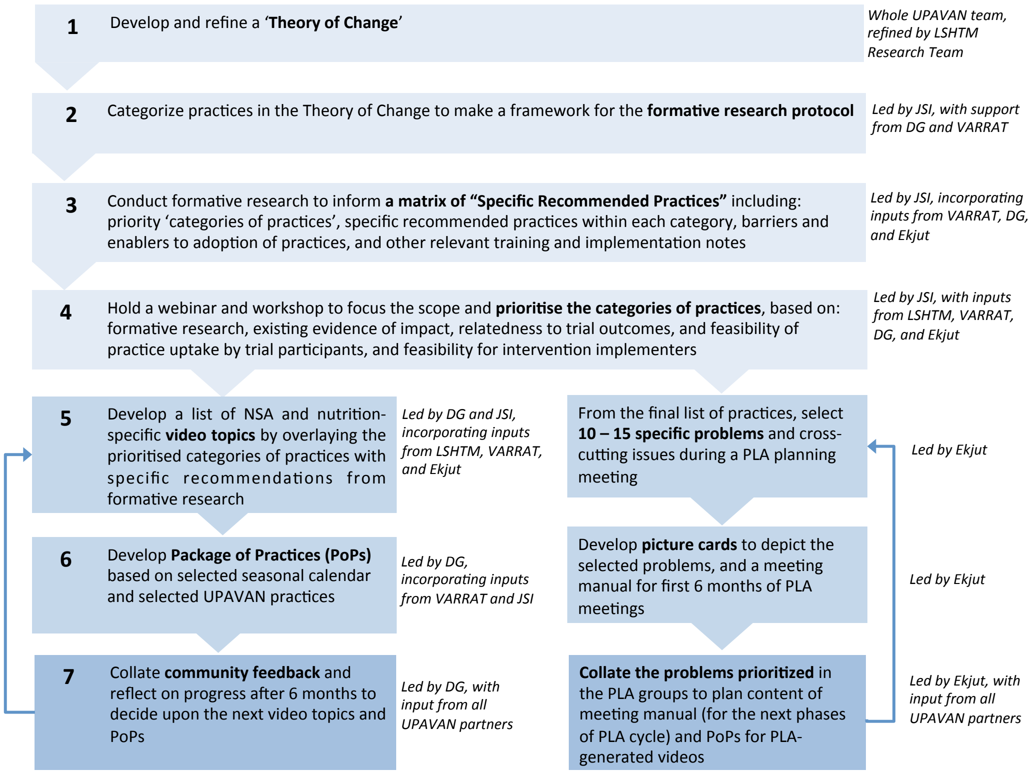

Figure 3 Process of prioritising NSA and nutrition-specific topics, practices and video or PLA content

The prioritization criteria for selecting video topics and PoPs are:

1. Adoption of the practice will **contribute along the pathway** to improve our primary outcomes (maternal BMI of non-pregnant women, and child dietary diversity).
2. The practice will have a **substantial impact** for those who adopt the practice. This considers how much of the population is already engaging in the practice, and how effective the practice itself is.
3. The practice is **feasible for target groups** to adopt and maintain. It is good practice to promote small, doable actions, and not ask for too many changes at one time.
4. It is **feasible for UPAVAN** to promote in terms of time, capacity, and human and financial resources. This considers feasibility for beneficiaries, and also the feasibility for project staff (given the resources available) to promote the practice with adequate coverage, equity, and quality.

We will prioritise practices that are more likely to be adopted and maintained over time. That is, those that are done once or infrequently, require little or no skill, show immediate positive results, are inexpensive to adopt, require little time, have little or no risk, fit existing social norms, can be done with existing resources and technology, require one person to do the job, and can be done in a single step.

3. Video dissemination

All videos will be disseminated in women's groups. More detail on community mobilization and group formation process is given in Section 7.

- Women's groups are typically formed of two Self-Help Groups (SHGs), or whatever combination is required to create an optimal group size of 20 to 25 members.
- There will be 5-6 women's groups, formed of 10-11 SHGs, per 1000 population.
- Since clusters comprise multiple villages and hamlets, care will be taken to ensure that hamlets are not excluded.

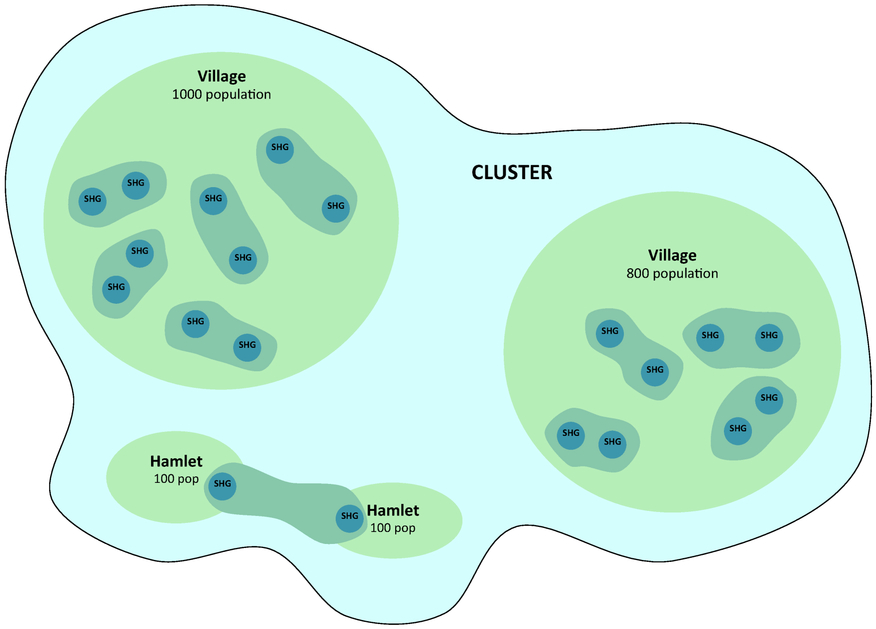

Figure 4 Illustration of the video dissemination platform, created by combining SHGs

Intended coverage:

- **At least one person per household attending fortnightly video disseminations**
- **All women in the 1000-day period attending fortnightly video disseminations**
- **5-6 women's groups for videos / 1000 population (10-11 SHGs)**

Videos will be disseminated by a trained VARRAT facilitator (a 'Community Support Person' (CSP)), and shown using low-cost, handheld pocket Pico projectors.

The videos use a range of styles to convey the messages, including testimonials from local community members who have adopted new or promoted practices, instructive videos, conversations with frontline workers (extension agents or AWWs), or (in arm 3) summary videos of previous meetings. If a new concept is to be introduced within the community, a government extension worker is also included in the video to increase the credibility of the video's message.

The group members collectively decide the date, time and venue of each video screening. At the screening, the CSP pauses the video at strategic points, encouraging the viewers to discuss and reflect on what they see in the video.

Videos produced in arm 1: Fortnightly nutrition-sensitive agriculture videos.

- *There is double the number of NSA videos in arm 1 compared with the other intervention arms.*
- *One of the NSA videos in each month will be shown in the other two intervention arms.*
- *The other NSA video (not shown in the other arms) will be on the same broad NSA topics but will cover different NSA micro-practices.*

Videos produced in arm 2: Monthly nutrition-sensitive agriculture videos plus monthly nutrition-specific videos

- *Ideally, CSPs should show one NSA video and one nutrition-specific video each month. In special circumstances (seasonal demand), 2 NSA videos or 2 nutrition-specific videos could be shown. However, over whole intervention period, an equal number of NSA and nutrition-specific videos must be shown.*

Videos produced in arm 3: Monthly nutrition-sensitive agriculture videos, plus 13 nutrition-specific videos informed by participatory approaches, as described below.

- *In arm 3, nutrition-specific video content will be informed by a participatory process of group discussion, problem solving, and priority setting. However, the videos will still be disseminated using the same platform of women's groups of combined SHGs.*

Harris-Fry H, *et al. BMJ Global Health* 2020; 5:e002384. doi: 10.1136/bmjgh-2020-002384

4. Participatory Learning and Action (PLA) in Arm 3

In arm 3, we will integrate a 'Participatory Learning and Action' (PLA) cycle approach, which is a capacity-building approach that enables group members to identify, understand, and implement feasible strategies to address problems in their community.

Groups will discuss and solve nutrition-specific issues that are relevant to them, find solutions, and implement strategies by sharing responsibilities, building group solidarity, using locally available resources, and using support from other community members.

This information from the group priority-setting and problem-solving will be used to create nutrition-specific videos. Although the PLA process will be used to generate PLA videos in slightly different ways for each phase (described below), the end result is that the PLA video content is driven by community priorities and ideas.

Since the NSA videos are also shown (in the same way as the other arms), women's groups will solve nutrition-specific problems whilst **making linkages with NSA topics**.

The same CSPs who show the nutrition-sensitive videos will also facilitate these PLA meetings support this process. As the CSPs will be trained in participatory methods for facilitating group discussions, **CSPs are required to incorporate participatory facilitation techniques** (demonstrations, storytelling, games, etc.) during the PLA-generated video disseminations. CSPs will brainstorm ideas and plan for these more participatory video dissemination discussions during the review meetings. These participatory techniques can also be used by the CSPs for the NSA video disseminations as well, but this is not required and may occur organically.

In arm 3, there are 2 group structures:

i. **Groups for viewing NSA and PLA-generated nutrition-specific videos** (as in A1 and A2) combine SHGs to give groups of 20-25 members (with 10-11 SHGs per 1000 population)
ii. **PLA groups for participating in the PLA meetings** (no videos shown), with 35-40 members per group (with 2 PLA groups per 1000 population). PLA groups will be demand-driven, so there may be more groups or larger groups in some clusters. We anticipate overlap in the group membership between PLA groups and SHG-based women's groups for video dissemination.

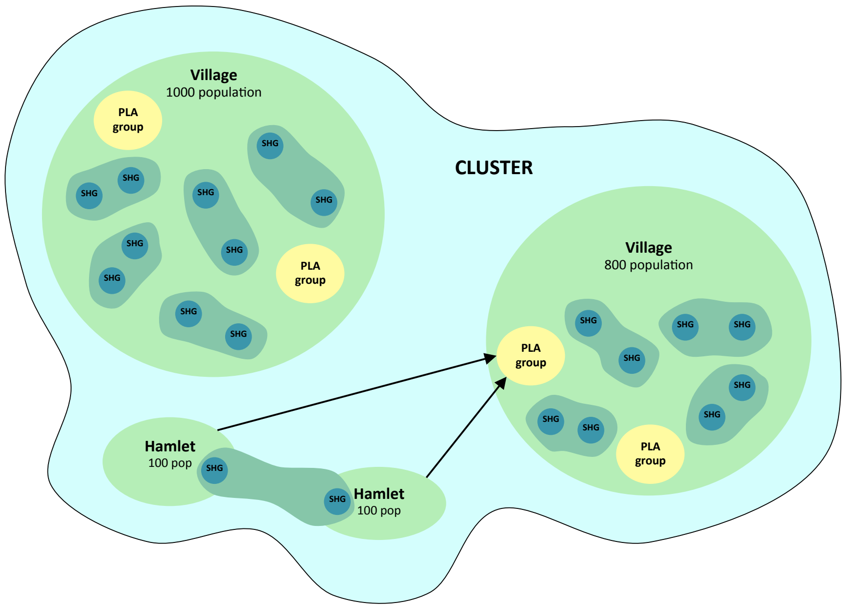

Figure 5 Illustration of the video dissemination platform, created by combining SHGs, with additional PLA groups

The PLA cycle comprises four phases:

1) **Identify and prioritise problems**

Meetings 1 to 5: *4 PLA meetings + 1 PLA-video + 5 NSA videos*
PLA group members will discuss local practices relating to a list of priority topics pre-selected by the study team on the basis of the matrix of recommended practices. The members will play a 'picture card game' to identify and discuss those selected problems. After 3 PLA meetings, a video summarizing the activities of the first three meetings will be disseminated to the wider community. In the last meeting of this phase, groups will focus prioritize problems and explore local practices and beliefs pertaining to the prioritized problems.

2) **Explore causes of prioritised problems, identify and prioritise strategies**

Meetings 6 to 10: *3 PLA meetings + 1 PLA-Video + 1 PLA-video with wider community + 5 NSA videos*
In the second phase, the members will explore the causes of the prioritized problems by listening to stories that will be created by the CSPs using local themes. The stories will illustrate how health, nutrition and hygiene problems are linked to underlying household, community level and health service related issues. The stories will also integrate components of NSA videos disseminated by the CSPs. Groups will discuss the underlying causes and solutions, and then decide which of problems they want to address and how. After prioritizing feasible strategies, there will be a video dissemination that summarizes the prioritized problems, strategies selected, and findings from the previous 3 meetings,

such as barriers that were commonly discussed among the groups. This video will be shared with the wider community to inform them about the PLA groups' strategies, and to gain support during implementation. At the end of the second phase, the PLA group members will organize a larger meeting of all stakeholders (involving other community members) to share the processes that they have gone through and the strategies they have prioritized.

3) Implement strategies

Meetings 11 to 27: *8 PLA meetings + 9 PLA-video + 17 NSA videos*
In the third phase, the PLA group members will implement and review the strategies they have decided upon. At this time, the CSP will also show videos on positive strategies for them to try at home, drawing from the formative work and matrix of recommended practices. The content of the videos will relate to the problems that are most commonly prioritized by the groups, and will also integrate emerging issues and barriers or enablers to behaviour change that arise from the strategy planning discussions.

Groups will choose when they would like to have a PLA meeting for a strategy, and when they would like to watch / discuss a video on the strategy. It is intended that there will be a final exposure of 8 PLA meetings and 9 video disseminations in Phase 3, but there may be some variation between groups if some strategies take more or less planning than others. At the end of the third phase, the PLA group members of neighboring villages belonging to one cluster will organize a larger meeting of all stakeholders to disseminate about their learning during the 3 phases of the PLA cycle.

4) Evaluate the process

Meetings 28 to 29: *2 PLA meetings + 1 video dissemination + 3 NSA videos*
In the fourth and final phase, the members will discuss the progress of their strategies and share achievements and difficulties encountered during the process of intervention. The participants will do a self-evaluation of what they have done well and where they could improve. The **way forward and the impact of the intervention** will be discussed with community members through video dissemination to the community towards the end of the PLA cycle. The content of this video will be based on a few testimonials, success stories, challenges faced by the community members in trying to implement and change practices, strategies employed for overcoming those challenges; and how they plan to take this learning ahead so that issues around nutrition can be addressed in the long run.

Harris-Fry H, *et al. BMJ Global Health* 2020; 5:e002384. doi: 10.1136/bmjgh-2020-002384

5. Home visits

In all arms, CSPs will conduct fortnightly** home visits to all women in the 1000-day period, to encourage and monitor the adoption of NSA or nutrition-specific practices that are discussed in the videos or PLA meetings.

CSPs will conduct home visits for the following reasons:

- Check knowledge recall of key messages and self-reported behavior using a Job Aid and Attendance and Home Visit form
- Reinforce key messages
- Create linkages between AWW/ASHA workers and beneficiaries and refer visibly ill or malnourished children, when required
- Mobilize beneficiaries for the next meeting/ video dissemination

Home visits will be conducted within 2 weeks of video screening after every video dissemination (or relevant (phase III) PLA meetings). Home visits should be done in a quiet, private place by the mediator and will last about 10-15 minutes. CSPs will use the attendance form to identify direct beneficiaries (i.e. 1000-day women). See protocol on Monitoring Information Systems for more details on how the information will be used.

During the home visit, CSPs will ask what the beneficiary liked the video or PLA meeting and what they remembered in a casual, open-ended conversation (without direct probing). At the end of the conversation, the CSP will ask about the key practice highlighted in the video and will probe whether they have adopted the practice and the reasons why. CSPs will record beneficiaries' recall of the key messages and adoption of behaviors on the home visit section of the attendance sheet, using a video or meeting-specific 'cheat sheet' job aid with correct answers. The job aid will clearly specify what the beneficiary must recall without probing for the CSP to give them a tick "yes." These job aids will be distributed to CSPs at review meetings. See dropbox for home visit job aid template.

Any qualitative feedback from the home visit will be recorded in the box at the back of the form and discussed during the review meeting.

All home visits should include the following steps:

1) Build rapport with the beneficiary (1000-day woman)
2) Knowledge recall: ask the beneficiary what she recalls from the video (without probing about specific topics/ points) and report this on the home visit form
3) Self-reported behaviours: ask verbatim the questions on self-reported behaviors related to the video, physically verifying if possible, and report adoption of practices on the home visit form.
4) Reinforce messages, clarify anything the beneficiary has misunderstood
5) When relevant, strengthen the link between beneficiaries and community health workers in their area (e.g. referrals to ASHA, AWW, and ANM for ill or malnourished children)
6) Encourage attendance at the next meeting

CSPs should carry along the attendance and home visit format and job aid for the particular video being checked.

** In arm 3, some PLA meetings will not require home visits because knowledge recall or adoption verification may not be applicable for the content of some meetings.

Harris-Fry H, *et al. BMJ Global Health* 2020; 5:e002384. doi: 10.1136/bmjgh-2020-002384

6. Frontline worker training

In all arms, including the control arm, frontline workers (ASHAs and AWWs) will be given **a 2-day training workshop** on maternal, infant and young child nutrition. This will be completed within the first 2 months of program implementation.

The main purposes of this training are:

- To promote engagement of frontline workers in the intervention.
- To strategically engage with government processes, which will be required in future during trial results disseminations, for engagement with policymakers in the future, and to promote scale-up.
- To provide some benefit to the control areas, which helps with data collection at endline, process evaluation, and monitoring of adverse events

Refer to the training protocol for more detail.

7. Women's group formation: community engagement, and mobilization of target beneficiaries

Groups will be formed and facilitated by VARRAT Community Support Persons (CSPs). After the newly recruited CSPs are oriented and inducted, they will **collect information about SHGs** in their respective villages and **set a time and date for video dissemination**. Typically, two self-help groups will be merged together to form one video-viewing women's group.

During this process, they will also **identify the households who are not part of any SHGs** and encourage them to: become a part of an existing SHG, form a new SHG, or form an informal video dissemination or PLA group. Where possible, women's groups will be formed by working with existing women's Self-Help Groups (SHGs, existing credit and savings groups); in other places with no existing SHGs, new groups will be formed. SHG formation will follow guidelines and register the groups officially, unless communities prefer a more informal mechanism.

At least one member in each household must be invited to attend.

On an ongoing basis, CSPs will encourage individuals to attend video disseminations during the home visits. In the PLA meetings in Arm 3, **PLA group members will also purposefully invite target or marginalized people** (1000-day women and people living in hamlets and remote villages).

- **The optimal group size for viewing videos is 20-25 individuals.**
- **The optimal group size for PLA meetings is 35 - 40 individuals**

A community launch event will be designed by VARRAT, Ekjut and DG staff, and will be executed by teams of CSPs (3-4 per team) in all intervention villages in late February/ early March 2017, prior to

Harris-Fry H, *et al. BMJ Global Health* 2020; 5:e002384. doi: 10.1136/bmjgh-2020-002384

intervention rollout. The purpose of these events is to introduce the community to the project and to generate interest.

The launch event is a 1-hour meeting in each village. Different pictures focusing on the concept of the first 1000-days will be displayed at the meeting venue for the participants to see and CSPs to explain. The CSP will give a brief about the project and details about what will happen in that village. The meeting will involve SHG representatives, village heads like ward members, Sarpanch, AWWs, ASHA, ANM, and other relevant people in the village. The meeting will be organised at a common place that is convenient for the village members.

Harris-Fry H, *et al. BMJ Global Health* 2020; 5:e002384. doi: 10.1136/bmjgh-2020-002384

8. Process of PoP and video development

a. Development of video topic and PoPs

JSI, VARRAT and Digital Green will jointly develop a calendar of video topics for the first 6 months (12 NSA and 6 MIYCN videos). The topics for these 18 videos will come from the formative research analysis, experience of VARRAT/DG in Keonjhar context, and insights from secondary data (NFHS-4 Odisha data, Ekjut's CARING trial in Keonjhar, etc.).

For the first video, JSI will develop a draft template for PoP development, and provide it to Digital Green for developing a storyboard and shooting one NSA video during a field visit by JSI (February 2017).

Digital Green will prepare the initial 18 PoPs on MIYCN (n=6) and NSA (n=12). The PoPs will be developed by DG/VARRAT but will be reviewed by JSI, at least during the first year of programming. After the first six months of review from JSI, a decision will be made about whether continued technical support is required and appropriate.

Half of the NSA videos will be the same across all arms.

JSI together with DG and VARRAT will decide on which NSA videos should be shown in A2 and A3 each month (given that there will be 2 videos produced for A1 every month, but only 1 shown in A2 and A3).

b. Joint Review of PoP

During PoP production, we will identify the categories of direct beneficiaries, key messages, and self-reported behavior to be tracked in the home visits (refer to section 5 and MIS protocol for more information on home visit process and how the information will be used). A maximum of 5 key messages and one self-reported behaviors or practice will be included. Once vetted by technical experts, these points will be incorporated into the video storyboarding and video production process.

The PoPs will be jointly reviewed by JSI, Digital Green, and VARRAT. Ronali will be responsible for facilitating joint reviews of PoPs.

Participants in the Joint Review of PoPs include:

- Digital Green (Ronali- facilitator, Meghan- M&E)
- At least 1 participant from VARRAT, and
- At least one technical content specialist from JSI (depending on whether the video topic is on NSA or nutrition-specific practices).

Initially, joint reviews will happen by email (or Skype when possible), before video production and storyboarding commences.

The final PoPs are required at least 1 month before the start of video dissemination to give time for storyboarding, video production, subtitling, and joint video review.

c. Video production

Local, trained filmmakers (Community Resource Persons, CRPs) from VARRAT make the videos, and one team of CRPs can produce two videos per month.

Using Digital Green's standard approach for developing videos, the video production process will be as followings:

i. Based on the PoPs given to them, the CRPs will develop the storyboards in Odia (local language), The storyboard will be reviewed by the DG Odisha team.
ii. CRPs identify actors, scout for suitable shooting locations, and shoot the video. Video production takes 7-9 days, starting from storyboarding to draft video.
iii. After the shooting is over, the clips are imported to editing software, and an 'editor CRP' will edit the video after inserting the key messages (non-negotiable knowledge recall points or behaviors) in the video as annotated points.

d. Joint review of videos

The draft video will be reviewed jointly by JSI and Digital Green, and optionally also by LSHTM and Ekjut. The initial 6 videos (3 nutrition-specific, 3 NSA) will be subtitled in English by Digital Green and uploaded in YouTube to enable the team to review and give comments or suggestions. Joint review of videos will be coordinated and facilitated by Ronali and Meghan and will ensure adherence to quality parameters outlined in a video review form (in Dropbox).

Feedback must be provided within 1 week of circulation.

Joint review of videos over Skype will occur within 1 week of video production initially, but may switch to written feedback alone to reduce workload on all partners. The video will be further edited by the CRPs and finalized after incorporating the feedback. Re-shooting will not be possible in all the cases, unless there is a major error in the technical content.

9. Video pre-testing

Our initial plan was to pre-test the first 6 videos (3 MIYCN, 3 NSA) in localities that are not part of the UPAVAN trial, such as villages that were part of the feasibility study. If required, the videos could be edited to incorporate community feedback. JSI has a video pre-testing form that we will use during pre-testing of the first few videos to collect community feedback. However, after the first month the team found that pre-testing was not feasible due to time constraints.

Instead, during the arm-wise fortnightly review meetings, the **CSPs will view the video** before dissemination and give their feedback. This feedback will be used **to pre-empt questions** that might arise during video dissemination, and to brief CSPs on possible responses that they could give.

Harris-Fry H, *et al. BMJ Global Health* 2020; 5:e002384. doi: 10.1136/bmjgh-2020-002384

a. Process of PLA-generated video production

In arm 3, the PLA process will determine the content of the nutrition-specific videos. This information will be fed from the groups to the Ekjut Project Leads, using meeting-specific forms that PLA supervisors use to document the groups' activities.

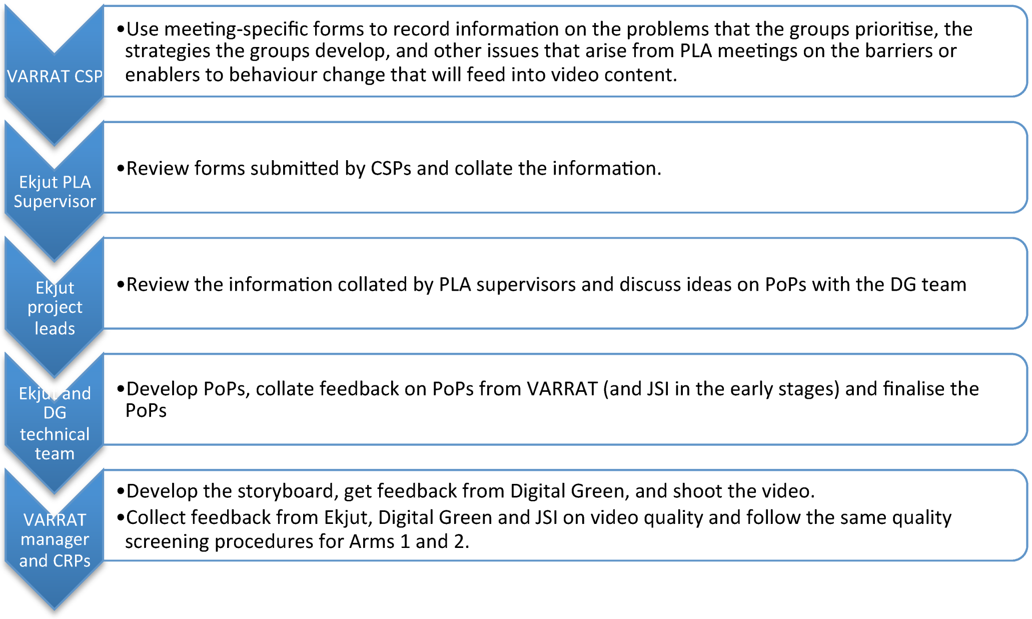

Figure 6 Process of developing PoPs and videos in Arm 3

In phase 1, after completion of 3 PLA meetings, there will be a video dissemination that will **summarize the activities** of the first three meetings. This video will be shared with the wider community to maximise participation in the next meeting when they will vote to prioritise the identified problems.

In phase 2, after prioritisation, there will be a video dissemination to summarize **problems that were prioritized, strategies selected, and barriers** that were commonly discussed among the groups in the 3 previous meetings. This video will be shared with the wider community to inform them about the strategies and gain support during implementation.

In phase 3, the content of the videos will relate to the **problems that were most commonly prioritized by the groups**, and will also integrate emerging issues and barriers or enablers to behaviour change that arise from their meetings. Groups will choose when they would like to have a PLA meeting or a video, but there will be a final exposure of 8 PLA meetings and 9 video disseminations in Phase III (there may be some variation if strategies vary in the level of planning required).

In phase 4, the **way forward and the impact of the intervention** will be discussed with community members through video dissemination. This video will contain few testimonials, success stories, challenges faced in trying to implement and change practices, strategies employed for overcoming those challenges; and how they plan to take this learning ahead so that issues around nutrition can be addressed in the long run.

10. Intervention delivery time plan

Video dissemination start date: 20 MARCH 2017

Table 2 Video dissemination and PLA meeting plan by month

Month	Arm 1: AGRI		Arm 2: AGRI-NUT		Arm 3: AGRU-NUT+PLA		
**2017**<br>***18 video disseminations (including breaks over 2 fortnights)***							
1 – Mar	NSA	NSA	NSA	Nutrition-specific	NSA	PLA meeting	*Phase I: Problem identification*
2 – Apr	NSA	NSA	NSA	Nutrition-specific	NSA	PLA meeting	
3 – May	NSA	NSA	NSA	Nutrition-specific	NSA	PLA meeting	
4 – Jun	NSA	NSA	NSA	Nutrition-specific	NSA	PLA video	
5 – Jul	NSA	NSA	NSA	Nutrition-specific	NSA	PLA meeting	
6 – Aug	NSA	NSA	NSA	Nutrition-specific	NSA	PLA meeting	*Phase II: Develop strategies*
7 – Sep	NSA	NSA	NSA	Nutrition-specific	NSA	PLA meeting	
8 – Oct	NSA	NSA	NSA	Nutrition-specific	NSA	PLA video	
9 – Nov	NSA	NSA	NSA	Nutrition-specific	NSA	PLA meeting	
10 – Dec	NSA	NSA	NSA	Nutrition-specific	NSA	PLA meeting (with video)	
**2018**<br>***20 video disseminations (allowing breaks of 4 fortnights)***							
11 – Jan	NSA	NSA	NSA	Nutrition-specific	NSA	PLA meeting	*Phase III: Act together (and continue learning)*
12 – Feb	NSA	NSA	NSA	Nutrition-specific	NSA	PLA video	
13 – Mar	NSA	NSA	NSA	Nutrition-specific	NSA	PLA meeting	
14 – Apr	NSA	NSA	NSA	Nutrition-specific	NSA	PLA video	
15 – May	NSA	NSA	NSA	Nutrition-specific	NSA	PLA meeting	
16 – Jun	NSA	NSA	NSA	Nutrition-specific	NSA	PLA video	
17 – Jul	NSA	NSA	NSA	Nutrition-specific	NSA	PLA meeting	
18 – Aug	NSA	NSA	NSA	Nutrition-specific	NSA	PLA video	
19 – Sep	NSA	NSA	NSA	Nutrition-specific	NSA	PLA meeting	
20 – Oct	NSA	NSA	NSA	Nutrition-specific	NSA	PLA video	
21 – Nov	NSA	NSA	NSA	Nutrition-specific	NSA	PLA meeting	
22 – Dec	NSA	NSA	NSA	Nutrition-specific	NSA	PLA video	
**2019**<br>**16 video disseminations *(allowing for breaks of 2 fortnights)***							
23 – Jan	NSA	NSA	NSA	Nutrition-specific	NSA	PLA meeting	*Phase III: Act together (and continue learning)*
24 – Feb	NSA	NSA	NSA	Nutrition-specific	NSA	PLA video	
25 – Mar	NSA	NSA	NSA	Nutrition-specific	NSA	PLA meeting	
26 – Apr	NSA	NSA	NSA	Nutrition-specific	NSA	PLA video	
27 – May	NSA	NSA	NSA	Nutrition-specific	NSA	PLA meeting	
28 – June	NSA	NSA	NSA	Nutrition-specific	NSA	PLA meeting	*Phase IV: Review and plan ahead*
29 – Jul	NSA	NSA	NSA	Nutrition-specific	NSA	PLA meeting	
30 – Aug	NSA	NSA	NSA	Nutrition-specific	NSA	Community meeting	
31 – Sep	NSA	NSA	NSA	Nutrition-specific	NSA	Community meeting	
32 – Oct	Community handover activities		Community handover activities		Community handover activities		

Harris-Fry H, *et al. BMJ Global Health* 2020; 5:e002384. doi: 10.1136/bmjgh-2020-002384

Table 3 Video or PLA meeting topics for the first year (2017 – 2018)

	Month	Arm 1: AGRI		Arm 2: AGRI-NUT		Arm 3: AGRI-NUT+PLA	
**1**	Mar	Spinach for nutrition	-	Spinach for nutrition	-	Spinach for nutrition	-
**2**	Apr	Benefits of chicken farming	Little gourd	Benefits of chicken farming	Hand-washing	Benefits of chicken farming	Introduction and understanding social inequities
**3**	May	Chicken farming, part 2	Summer plough	Chicken farming, part 2	Diarrhea	Chicken farming, part 2	Understanding underlying causes of under nutrition
**4**	Jun	Rice, part 1	Rice, part 2	Rice, part 1	Rice, part 2	Rice, part 1	Rice, part 2
**5**	Jul	Pot manure	Rice, part 3	Pot manure	Rice, part 3	Identifying the problems related to MIYCN and Hygiene	Rice, part 2
**6**	Aug	Ridge gourd for nutrition	-	1000-day	-	Sharing of problems and summary of findings of the first three meetings with the wider community	-
**7**	Sep	Kitchen garden	Carrot	Kitchen garden	Nutrition during pregnancy and lactation	Kitchen garden	Prioritizing of problems

	Month	Arm 1: AGRI		Arm 2: AGRI-NUT		Arm 3: AGRI-NUT+PLA	
**8**	Oct	Cow pea	Composting	Cow pea	Adding an extra meal during pregnancy	Cow pea	Understanding the causes and effects of 2 prioritized problems
**9**	Nov	Drying and storage	Spending decisions	Drying and storage	Spending decisions	Drying and storage	Spending decisions
**10**	Dec	Mushroom cultivation (oyster)	Cultivation of different species of Amaranthus	Overcoming concerns about eating less during pregnancy	Cultivation of different species of Amaranthus	Understanding barriers and opportunities for the strategies, and prioritizing feasible strategies community (community viewing)	Cultivation of different species of Amaranthus
**11**	Jan	Overarching NSA instructive video	Chicken (testimonial, vaccination, and penning)	Exclusive breastfeeding	Chicken (testimonial, vaccination, and penning)	Video dissemination - Sharing of prioritised strategies and summary of findings with the wider	Chicken (testimonial, vaccination, and penning)
**12**	Feb	-	Mushroom cultivation (paddy straw)	-	Diet of breastfeeding women	-	Accountability and sharing of responsibilities

Harris-Fry H, *et al. BMJ Global Health* 2020; 5:e002384. doi: 10.1136/bmjgh-2020-002384

1. Operational steps for trial Integrity

Refer to the UPAVAN research protocol for research details including inclusion and exclusion criteria for the participation in the study.

Consent

Before starting the intervention, written (or verbal, and signed by witness) consent must be requested from all village leaders in each of the clusters selected for the study. Consent must not be coerced and village leaders must be given sufficient time to make their decision on whether to participate. Intervention participants will not receive any stipend or gift for participating.

Consent forms must be stored in a secure, locked cupboard. Villages are free to withdraw their consent at any time, and **any withdrawn consent must be reported within 24 hours to the UPAVAN trial manager.**

Blinding

The trial statistician and principal investigator are 'blinded' to the allocation, meaning that they should not know the allocation of clusters to trial arms. **Implementation partners should ensure that the full list of clusters and their allocation is confined to the implementation team members.**

Contamination

Clusters shall not be exposed to any of the intervention components (video disseminations, PLA meetings or home visits) from the other treatment arms. Although participants may move between clusters (e.g. due to typical migration patterns), the implementation at the cluster level should be contained.

In practice, this means that:

- Control clusters should not receive any intervention components apart from the 2-day training to frontline workers that is provided to all arms;
- A1 clusters should not receive any nutrition-specific videos, PLA meetings, PLA-generated videos, or home visits on nutrition-specific practices;
- A2 clusters should not receive more than half of A1's NSA videos, or any PLA meetings or PLA-generated videos;
- A3 clusters should not receive more than half of A1's NSA videos, or any nutrition-specific videos that were not generated through the PLA process.

To ensure this, we have the following requirements (Table 4)

Table 4 Requirements to ensure no contamination between arms

**No or minimised crossover of staff between arms**	• The video content will be reviewed by DG and JSI subject matter experts to ensure that there is no overlap of nutrition-specific content in A1, or PLA-related content in A1 or A2.<br>• After training, arm coordinators, supervisors and CSPs will be allocated to work in a specific intervention arm, and cannot be reallocated to a different intervention arm at any point in the trial.
**Equal competence of staff between arms**	• CRPs (Community Resource Providers) will make videos for use across all 3 intervention arms, ensuring consistency in quality of video production.<br>• All CSPs will participate in the same trainings on NSA, nutrition-specific topics, and video dissemination, prior to intervention roll-out in all 3 arms.<br>• CSPs will be assessed with a post-training test, and refresher training provided as needed (refer to training protocol)<br>• Only CSPs and PLA supervisors in arm 3 will be trained on PLA facilitation techniques.
**NSA intervention component is the same in all arms**	• Half of the NSA videos must be the same across all three intervention arms<br>• The initial set of NSA and nutrition-specific priority practices, along with a dissemination calendar, will be decided upon prior to intervention roll-out so that the same videos are shown across clusters within an intervention arm. However, the process of prioritization will be iterative, based on community feedback and interests from the first 6 months of roll out.
**Monitor external programs**	• Other projects on agricultural technologies, nutrition-sensitive agriculture and MIYCN should not be introduced by VARRAT.<br>• When possible, the trial manager should liaise with external organizations to prevent the implementation of projects disproportionately between arms.<br>• The trial manager is also responsible for monitoring and documenting if other organizations introduce such projects. This exposure will be assessed in the process evaluation.

Adverse events

Effort must be made to ensure that participants will also not be subjected to any harm by participating in the trial, and any unexpected adverse events must be reported to the trial manager within 24 hours. For more information, refer to the adverse events plan.

Compliance

Quality Assurance, using Monitoring Information Systems, will ensure that interventions are delivered to a high standard and meet intended coverage, dosage, and quality targets. Refer to the Quality Assurance Plan for more information.

Appendix 1

UPAVAN operational definitions and acronyms

AGRI	Also called 'Arm 1'. This is the intervention arm that receives an agricultural extension intervention of low-cost participatory videos and facilitated discussions through self-help groups on nutrition-sensitive agriculture.
AGRI-NUT	Also called 'Arm 2'. This is the intervention arm that receives an agricultural extension intervention of low-cost participatory videos and facilitated discussions through self-help groups on nutrition-sensitive agriculture (half of AGRI) combined with maternal, infant and young child nutrition behaviour change through the same low-cost participatory videos and facilitated discussions.
AGRI-NUT+PLA	Also called 'Arm 3'. This is the intervention arm that receives an agricultural extension intervention of low-cost participatory videos and facilitated discussions through self-help groups on nutrition-sensitive agriculture (half of AGRI), combined with participatory learning and action (PLA) cycle meetings to discuss on nutrition-specific topics and to generate videos topics for future video disseminations.
ANM	Auxiliary nurse midwife - village-level female health worker traditionally focussing on midwifery, but now also providing immunisations and having a broader and maternal and child health remit.
ASHA	Accredited Social Health Activist – female community member who acts as the interface between the community and the public health system
AWW	Anganwadi worker – female community worker who provides basic health care activities, including contraceptive counselling and supply, nutrition education and supplementation, and pre-school activities.
COCO	Connect Online Connect Offline – Data collection and monitoring platform to support the monitoring information system
CSP	Community Support Person – local community person employed by VARRAT to disseminate videos and conduct home visits. Other roles described in UPAVAN > Operational Protocol > UPAVAN_OP004_Roles and Responsibilities.
CRP	Community Resource Providers – local community person employed by VARRAT to produce videos. Other roles described in UPAVAN > Operational Protocol > UPAVAN_OP004_Roles and Responsibilities.
Micropractice	A single action or behaviour that a person can implement or adopt (e.g. eat one egg a day)
MIS	Monitoring Information System
MIYCN	Maternal, infant and young child nutrition
NSA	Nutrition-sensitive agriculture. Nutrition-sensitive agriculture refers to agricultural approaches which incorporate specific nutrition goals. These

	activities and interventions aim to improve nutrition through women's empowerment by addressing the underlying causes of undernutrition--physical, economic, and socio-cultural access to nutritious food year-round; and adequate resources for health, WASH, and care at individual and household levels. Nutrition-sensitive agriculture does no harm to human or environmental safety. Nutrition-sensitive agriculture is distinct from nutrition-specific interventions which address the immediate determinants of undernutrition – consumption of adequate food and nutrient intake; practice of optimal health, WASH and care behaviors; and reduction disease burden.
PLA	Participatory Learning and Action: This involves a four-phase participatory process facilitated by a trained facilitator, in which women's groups collectively decide priority actions, and try to organize activities accordingly. The cycle is structured as follows: Phase 1: identify and prioritize problems; Phase 2: plan activities; Phase 3: implement strategies to address the priority problems; Phase 4: assess the activities
PoP	Package of Practice – the messages and practices included in a video
Practice	A group of actions or behaviours (micropractices) that a person can implement or adopt (e.g. diversify diet) and that relate to intended trial outcomes (such as dietary diversity)
QA	Quality assurance
SHG	Self-help group – Indian self-governed, peer controlled village committee for savings and credit
TMG	Trial Management Group: Team of representatives from each partner organisation that has monthly meetings.
TSC	Trial Steering Committee: External committee for monitoring trial progress, protocols and harms.
UPAVAN	Upscaling Participatory Action and Videos for Agriculture and Nutrition

Appendix 2

UPAVAN Monitoring Information System and protocol to ensure quality and adherence to implementation protocol

Contents

1. Monitoring information system (MIS) 2
    a. Video dissemination and PLA meetings: attendance/ coverage 2
    b. Home visits: Knowledge recall and self-reported behaviours 3
    c. UPAVAN COCO 3
    d. Review meetings 4
2. Quality Assurance (QA) 6
    a. PoP and video quality review 6
    b. Field level QA: Mediation observation and home visit cross-verification 7
    c. COCO quantitative data review 8
    d. Review meeting qualitative data review 10
    e. HR capacity and post-training tests 11

Harris-Fry H, *et al. BMJ Global Health* 2020; 5:e002384. doi: 10.1136/bmjgh-2020-002384

1. Monitoring information system (MIS)

The same MIS will be used across the three intervention arms to track coverage and attendance, and to monitor progress and quality of the video dissemination, PLA meetings, and home visit components of UPAVAN.

a. Video dissemination and PLA meetings: attendance/ coverage

The primary source of coverage data is collected on Attendance and Home Visit forms, as illustrated in Figure 1**.** CSPs complete the first portion of these forms (left-hand side) at the video dissemination or PLA meeting, to record attendee name, husband/ father's name, age, gender, whether they fall into the 1000-day category (pregnant woman, mother of child 0-6 months, mother of child 6-23 months), and attendee's signature.

UPAVAN ATTENDANCE AND HOME VISIT FORM

Type of venue: ● AWC ● School ● Open Space ● Community Hall ● Other	Village:	Block and District:
● NSA video ● MIYCN video ● PLA video ● PLA meeting	Video name/Meeting topic:	
CSP name:		Frontline worker present? ● ANM ● ASHA ● AWW
Date of dissemination/meeting:		Dissemination/meeting start time: end time:

Name of group(s): 1.________ 2.________ 0. Beneficiary who does not belong to any group

**** Fill this section during video dissemination/PLA meeting ⇩**

S. No.	Name of beneficiary	Father / husband name	Age	Gender	Group code	Put ✓ for all that apply to the beneficiary			Signature of beneficiary
						Pregnant woman	Mother of a child up to 6 months	Mother of a child 6 months to 2 years	
1									
2									
3									

**** Fill this section during home visit of all women in 1000 days ⇩**

Date of home visit	Has the beneficiary recalled the knowledge recall point? Put ✓/ ☒					Is the beneficiary practicing the behavior? Put ✓ / ☒ / —		Signature of beneficiary
	1	2	3	4	5	1	2	

Figure 1 UPAVAN Attendance and Home Visit form

We identify all attendees who are in the 1000-day period because they are 'direct beneficiaries' of the intervention. That is, the intervention is targeted at women and their children in this period. **All women identified as being in the 1000-day period on the attendance form will receive a home visit**. We also want to know what type of 1000-day women they are (pregnant woman, mother of child up to 6 months, mother of child aged 6 months to 2 years) to know what type of women we are reaching. Regardless of which video we show or PLA meeting content, all women in the 1000-day period will receive home visits.

The same attendance and home visit form will be filled out by the CSP during the video disseminations and PLA meetings in the three intervention arms (A1, A2, and A3). CSPs are encouraged to fill out as much of the form prior to the meeting as possible, to avoid wasting time during facilitation. Rigorous training and practice in using the forms will be provided as part of the video dissemination training for CSPs. The form is a slightly modified version of the USAID project attendance and home visit form, and it will be entered into the 'Connect Online, Connect Offline' (COCO) system, specifically tailored for UPAVAN.

The attendance and home visit format can be found in the UPAVAN Dropbox:

UPAVAN > Monitoring – MIS >Attendance and Home Visit Form

Harris-Fry H, *et al. BMJ Global Health* 2020; 5:e002384. doi: 10.1136/bmjgh-2020-002384

b. Home visits: Knowledge recall and self-reported behaviours

At the home visits, CSPs will collect individual-level data on **recall of key messages and self-reported behaviours** and record this on the second half (right-hand side) of the Attendance and Home Visit form. Home visits will be conducted after every video dissemination in all three arms (NSA, MIYCN, or PLA video) and after every PLA meeting in Phase III in arm 3. CSPs will conduct home visits to all 1000-day women who have attended the video dissemination/ PLA meeting. The same home visit approach and formats will be used consistently in all intervention arms (A1, A2, A3). Each home visit should take between 10-20 minutes.

All home visits should include the following steps:

1) Build rapport with the beneficiary (1000-day woman)
2) Knowledge recall: ask the beneficiary what she recalls from the video (without probing about specific topics/ points) and report this on the home visit form
3) Self-reported behaviours: ask verbatim the questions on self-reported behaviours related to the video, physically verifying if possible, and report adoption of practices on the home visit form.
4) Reinforce messages, clarify anything the beneficiary has misunderstood
5) When relevant, strengthen the link between beneficiaries and community health workers in their area (e.g. referrals to ASHA, AWW, and ANM for ill or malnourished children)
6) Encourage attendance at the next meeting

CSPs should carry along the attendance and home visit format and job aid for the particular video being checked.

See below for fortnightly targets for home visits.

Table 1 Arm-wise plan for home visits

Arm	Home visits
Arm 1	After every video dissemination (2 per month/direct beneficiary)
Arm 2	After every video dissemination (2 per month/direct beneficiary)
Arm 3	After every video dissemination (NSA or PLA video) + after PLA meetings in Phase III

c. UPAVAN COCO

Digital Green's COCO (Connect Online | Connect Offline) system will form the backbone of the monitoring system for UPAVAN. COCO is a web-based application that can be deployed without the need to download or install software. It makes data collection resilient to intermittent or limited internet connectivity with the ability to seamlessly toggle between online and offline modes while entering or viewing data for uninterrupted usage. COCO runs on modern web browsers on any device – desktops, laptops, tablets and smartphones.

UPAVAN COCO has been tailored by Digital Green's technology team based on the demands of this project. This includes additional fields (presence of health worker and venue) for video disseminations

and home visits (knowledge recall and self-reported behaviours), as well as inclusion of PLA meetings. Villages, CSP names, and group names have been uploaded into UPAVAN COCO.

Arm coordinators will be trained as data entry operators and will input video specifics in the "video" section of COCO after each video POP is reviewed and approved. All of the SHG members will be added to the "SHG" section of COCO on a one-time basis (this doesn't need to be repeated after each dissemination). The paper-based attendance and home visit form data will be entered into separate "screening" and "adoption" sections in COCO after each review meeting. Data entered in COCO will be analyzed by the DG MERL program manager every two months, as described in detail in the *Quality Assurance* section of the document.

Data entry timeline: For each video or PLA meeting, we will follow the timeline in Figure 2:

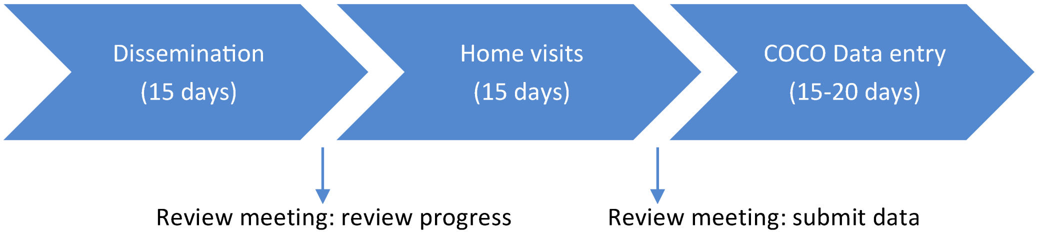

Figure 2 Data entry timeline for a given video or PLA meeting

Dissemination of one video or PLA meeting will happen over the course of one fortnight, followed by a review meeting to discuss progress and plan for the next fortnight. In the following fortnight, CSPs will conduct home visits pertaining to that video. In the following review meeting, CSPs will submit all data Attendance and Home Visits forms related to that video. COCO data entry for that video or PLA meeting will proceed for the next 15-20 days. Thus, data entry lag from video dissemination to data entry in COCO is 45-50 days in total.

d. Review meetings

VARRAT project coordinator will coordinate **1 review meeting per arm per fortnight** (1 meeting per video dissemination/PLA meeting) for a total of 6 review meetings per month, with support from the respective arm coordinators in that arm, to:

- Review last month's progress in terms of disseminations/ PLA meetings, and home visits (quantitative data)
- Discuss participants' feedback during disseminations and/ or home visits (qualitative field on attendance and home visit forms)
- Plan the upcoming month's activities and workload for each CSP
- Discuss and role play for the next month's videos, including pre-empting questions that might arise and briefing the CSPs on some possible responses.

NOTE: the project coordinator should try to encourage CSPs to bring as much qualitative insights from their experiences with disseminations/meetings and home visits as possible. These insights (particularly

about barriers to adoption) will help other CSPs in problem solving in the future and will inform video production moving forward.

Review meetings will be conducted arm-wise. Meetings will be a full daylong activity and will be located in a location most convenient for all CSPs in that intervention arm.

The following people will attend each meeting:

- All CSPs in that arm
- At least 1-2 CRPs (to collect feedback on videos),
- 1 arm coordinator
- VARRAT project coordinator
    - NOTE: Where possible, the review meetings for each intervention arm should not be conducted on the same day, so the project coordinator can be present at all meetings.
- VARRAT supervisors (all arms) and PLA supervisors (arm 3)
- When possible, one staff member from DG (and Ekjut for arm 3)

The arm coordinator, together with the project coordinator, will be responsible for documenting the feedback from the CSPs at every review meeting. A review meeting format has been developed to document the review meeting. The project coordinator is responsible for completing the form, translating into English, and sharing with Digital Green. Review meeting documentation will be filed arm-wise in the UPAVAN Dropbox after each review meeting by the Project coordinator

UPAVAN > Review meetings>armX

Digital Green's M&E manager and LSHTM will review the review form documentation every 4 months and report back during the implementation partners meeting with feedback.

Harris-Fry H, *et al. BMJ Global Health* 2020; 5:e002384. doi: 10.1136/bmjgh-2020-002384

2. Quality Assurance (QA)

Quality assurance for UPAVAN includes the following components:

- PoP and Video quality review
- Video mediation/ PLA meeting observation
- Home visit cross-verification
- COCO quantitative data review
- Review meeting qualitative data review
- HR capacity and post-training tests

a. PoP and video quality review

A process for POP and video quality review has been developed to ensure that quality parameters are consistently assessed. See operational protocol on interventions (UPAVAN_OP001_Interventions) for more detail on the PoP and video development and review process.

Ronali will be responsible for facilitating joint reviews of PoPs. Participants in these joint reviews include: Ronali (facilitator), Meghan, VARRAT (Shibananth), and technical content specialists from SPRING/JSI (depending on the video topic - NSA or MIYCN). Video review forms will be used as a guide for reviewing the video quality. Only those videos passing joint review will be incorporated into the DG video library in COCO and utilized as part of the UPAVAN intervention. Video review format and filled forms (NSA, MIYCN, and PLA) can be found in the UPAVAN Dropbox

UPAVAN> QA> Video Review and Pre-testing QA

The video will be further edited by the CRPs and finalized after incorporating the comments received on video quality and content. Re-shooting will not be possible in all the cases, and will only occur if there are major errors in the content.

For the first few videos, **we will pre-test the videos in villages that are not enrolled in UPAVAN to avoid contamination**, such as villages that were part of the feasibility study. Pretesting will be conducted different SHG groups for each video) to get community perspectives and feedback on the messages in the videos, and to assess whether they can relate to and understand the messages. SPRING/JSI has developed a video pre-testing form that we will use during pre-testing of the first few videos to collect community feedback. If required, the videos will be edited to incorporate the community feedback. The video pre-testing form can be found in the UPAVAN Dropbox

UPAVAN>QA> Video Review and Pre-testing QA

Update: Following this initial round of pre-testing, we found that pre-testing was not feasible for all videos. Instead, CSPs will view the upcoming video during their arm-wise fortnightly review meeting, and will give their thoughts, questions, and feedback. Collectively, the team (CSPs, supervisor, and arm coordinator) will discuss and pre-empt the sorts of questions that might arise during the video dissemination, and will discuss possible responses that the CSPs could provide during dissemination in these scenarios.

b. Field level QA: Mediation observation and home visit cross-verification

Mediation observation involves observing a CSP conduct a video dissemination to evaluate the quality of their facilitation skills, equipment handling, knowledge of the video topic, and form-filling ability. DG has a 'QA COCO' system for inputting these data. VARRAT staff (arm coordinators and project coordinator) will be primarily responsible for conducting mediation observations. However, DG staff will also monitor how these QA visits are going on a monthly basis (see exact targets below).
See UPAVAN Dropbox for mediation observation format:

UPAVAN> QA> Mediation observation QA> video disseminations

PLA meeting observations will be conducted qualitatively, using PLA meeting observation formats. QA of PLA meetings will be conducted by the Arm 3 Coordinator only.
See UPAVAN Dropbox for PLA meeting observation format:

UPAVAN> QA> Mediation observation QA> PLA meetings

CSPs are given a letter grade (A, B, or C) as part of the mediation observation format. CSPs scoring a 'B' grade or below are provided with a capacity building plan to ensure their further improvement. See HR capacity and post-training tests section below for further details.

Home visit cross-verification involves cross-verifying the knowledge recall and self-reported behaviour data collected by the CSPs during their home visits. Cross-verification is conducted on a random selection of 1000-day women who had been previously visited by the CSP to compare the knowledge recall and self-reported practice or behaviour with the responses collected by the CSP. VARRAT staff (arm coordinators and project coordinator) will conduct home visit cross verifications, and Digital Green (all arms)/ Ekjut (arm 3) will go along with VARRAT staff for random cross-verifications to ensure quality of these QA checks. Home visits will occur the fortnight following the dissemination/ PLA meeting. Thus, home visit cross-verification will occur in the 2nd fortnight (1 month) after the dissemination/ PLA meeting.

Home visit cross verification format can be found in the UPAVAN Dropbox:

UPAVAN> QA> Cross verification QA

The cross-verifier should carry the cross-verification form, the relevant job aid, and the home visit form that was previously completed by the CSP for that respondent.

Individuals conducting the home visit cross-verification should use the same methodology as the CSP uses for the home visits. This includes (1) building a rapport with the beneficiary; (2) asking what the beneficiary recalls from the video (without any probing of the specific video content); (3) asking the self-reported behaviour question(s) exactly as written on the job aid; (4) reinforcing any key messages or points the beneficiary didn't recall.

All mediation observation and home visit cross verification data will be entered into QA COCO by the arm coordinators/project coordinator on a monthly basis.

This field-level QA (mediation observation and home visit cross-verifications) will follow a 3-month cycle in the initial phase. Over three months, all clusters/CSPs will be reviewed. After the first 3-month cycle, the system for QA will be evaluated and modified if necessary.

Table 2 Plan for fortnightly QA checks

**Person conducting QA check**	**Mediation/ PLA meeting observations**	**Home visit cross-verifications****
VARRAT Arm Coordinators (per arm)	9(3)	18 or 12**(6)
VARRAT Project Coordinator (per arm)	3 (1)	6 or 4**(2)
*Digital Green staff**	*1*	*3*
*Ekjut staff**	*1*	*3 or NA*
Total (fortnightly)	**12**	**24 or 18****
Total (monthly)	**24**	**42**
Total (3 month cycle)	**72**	

* QA checks by DG and Ekjut staff are to check the quality of QA visits conducted by VARRAT and are not included in the fortnightly/monthly QA totals
**Every other fortnight, PLA meetings instead of video disseminations will occur in arm 3. For PLA meetings in Phase I, II, or IV, home visits and home visit cross-verifications are not required.

c. COCO quantitative data review

Attendance and home visit form data will be entered into COCO by the data entry operators (arm coordinators) for all three arms on a monthly basis with about a 45-50 day data lag (as shown in Figure 2). These data will give quantitative insights into the coverage the intervention is achieving, including the types of beneficiaries we are reaching (women in the 1000 days, SHG members, age, gender, etc.), the length of disseminations/ PLA meetings, and insights into knowledge recall and self-reported behaviour of the beneficiaries.

Digital Green **M&E manager will analyze the COCO data once in two months** to provide feedback on CSP performance, overall intervention progress, and to identify extreme outliers in CSPs, clusters, and video viewership, as summarised in Table 3. The Digital Green M&E manager will analyze COCO data and share an oral and powerpoint report of this preliminary data analysis at the bi-monthly implementation partners meetings (attended by DG, VARRAT, Ekjut, and LSHTM trial manager). This presentation will form the basis for discussions and trouble-shooting during this meeting. A written report will be shared with the rest of the UPAVAN team, including the data, analysis and action points decided upon during the implementation partners meeting.

Table 3 COCO data review – provisional parameters for tracking implementation

Indicator Category	Parameters
Implementation progress (achievement vs targets- see full table below)	- Videos produced<br>- Videos uploaded<br>- Video disseminated<br>- Disseminations conducted, arm-wise and video-wise<br>- Home visits conducted, arm-wise and video-wise<br>- Average home visits conducted/dissemination, arm-wise and videos
Outliers in implementation	- CSPs (average # disseminations, average # home visits, average #knowledge recalls, average percentage with self-reported behaviour)<br>- videos (average # disseminations, average # home visits, average #knowledge recalls, average percentage with self-reported behaviour)<br>- Duration of dissemination/meeting
Time trend for implementation	- Changes in number of videos disseminated, home visits, and averages
Quality Assurance progress	- Mediation observation, arm-wise<br>- Home visit cross verification, arm-wise<br>- CSP review and planning meetings, arm-wise
Average SHG attendance, arm-wise	- identification of SHGs with notably low or high attendance<br>- Number of attendees as a proportion of the number of households per cluster, compared to the target of one attendee per household.<br>- Proportion of direct beneficiaries (1000-day women) attending groups, and identification of groups with very high or low proportions of direct beneficiaries;
Missing data	- By arm, CSP, and video
Inconsistencies in self-reported behaviours by CSPs	- E.g. breastfeeding by woman without a child

These indicators may change after preliminary analyses.

Harris-Fry H, *et al. BMJ Global Health* 2020; 5:e002384. doi: 10.1136/bmjgh-2020-002384

Targets for implementation are given in Table 4.

Table 4 Fortnightly targets for implementation

Area	Indicator	Arm 1	Arm 2	Arm 3	Total
**Overall Implementation**	Videos produced[1]	1	1	1	1-3
	Videos uploaded and subtitled	1	1	1	1-3
	Videos disseminated/PLA meetings	1	1	1	1-3
	Disseminations (PLA meetings conducted)[2]	281	269	290 (110)	840 (660)
	Attendance	X	Y	Z	X+Y+Z
	Home visits conducted	3447	3447	3447 (0)	457 (307)
	Average home visits per dissemination conducted	14	14	14	
**CSP-wise Implementation**	CSPs	23	22	23	68
	Videos disseminated/ PLA meetings	1	1	1	1-3
	Disseminations / PLA meetings conducted (TBC)	12	12	13 (5)	37 (29)
	1000 day women attending dissemination/PLA meeting	150	157	150	457
	%1000 day women of total attendance	*Determine based on data from CSPs/AWW on village composition*			
	Home visits conducted	150	157	150	457

CSPs will also collect data on the number of 1000-day women in their assigned cluster or villages from AWWs every two months and submit to the project coordinator. The project coordinator will triangulate these data with attendance data during the review meetings to assess gaps in coverage and find ways to address these gaps.

d. Review meeting qualitative data review

The respective arm coordinator, with support from the project coordinator, will be responsible for documenting the feedback from the CSPs at every review meeting. A format for documentation has been developed (see UPAVAN Dropbox: UPAVAN > Review Meetings) and the project coordinator is responsible for ensuring the form is filled, translated into English, and shared with Digital Green. All review meeting documentation will be upload, arm-wise in the Dropbox under: UPAVAN > Review Meeting s> armX. Digital Green's M&E manager and LSHTM trial manager will also monitor the review meeting documentation every 4 months and include in the oral report/presentation during the bimonthly implementation partners meeting.

The review meeting documentation will help to inform the intervention including: planning for new video topics; CSPs requiring additional handholding; and major problem clusters. The insights from this documentation will also be incorporated into the process evaluation.

Appendix 3

UPAVAN operational plan for trainings

Contents

1. Technical content training for CSPs, CRPs, and Supervisors - NSA and MIYCN 1
2. Technical content training for AWWs and ASHAs (MIYCN)..2
3. Video dissemination, home visit, and community mobilization training for CSPs 2
4. PLA training for CSPs involved in A3 of the UPAVAN project 3
5. Data entry operator training..4

Training is a crucial precursor to UPAVAN intervention rollout, providing implementers with the base knowledge and skills to effectively complete their responsibilities. Training helps to ensure the CSPs and CRPs are all on the same page, conducting the intervention consistently across their clusters and interventions arms.

Most training will be delivered to the CSPs, who implement the intervention at the field level. CRPs responsible for developing the videos across all three intervention arms have already been trained in video production. However, they will be called to attend the technical content trainings, as this material is directly relevant for video production.

In all intervention arms (including the control), AWWs and ASHAs will also be trained on maternal, infant and young child nutrition, to ensure consistency of messages across formal health services and the intervention, as well as to foster buy-in to the intervention, as often these frontline health workers are members of SHGs and featured in nutrition-specific videos.

Trainings will commence in Feb 2017 and run to the start of March when intervention roll-out begins.

1. Technical content training for CSPs, CRPs, and Supervisors - NSA and MIYCN

Technical content training for CSPs, CRPs, and Supervisors will run for 14th to 21st February and will comprise 6, 2-day sessions; 3 sessions on NSA and 3 sessions on MIYCN. The training program will be designed by the JSI team based on their findings from the formative research conducted in Keonjhar in October 2016. The training packages will be developed by JSI and translated by DG into Oriya for all technical content trainings.

JSI will conduct a Training of Trainers (ToT) for NSA and MIYCN trainings and will be present to closely monitor trainings for CSPs and CRPs. All trainings will be facilitated in Odia by the VARRAT and DG team of 3 trainers on MIYCN/NSA. Trainings will be conducted in the same manner for all sessions to ensure a uniform skills and knowledge base across frontline workers, regardless of intervention arm. Trainings will be participatory and contextualized to Keonjhar’s agriculture, health and nutrition issues. Training methodology will include activity-oriented sessions like group discussions, role plays, picture cards, and case studies.

Harris-Fry H, *et al. BMJ Global Health* 2020; 5:e002384. doi: 10.1136/bmjgh-2020-002384

Each NSA or MIYCN training is two days, and will have a maximum of 30 people per training. All trainings will be conducted at VARRAT field office in Keonjhar. Each participant will get MIYCN training before NSA training.

All CSPs will be assessed by a post-training test to help guide the trainers in understanding which technical content requires additional focus and attention and help identify CSPs requiring more support. Test results will be uploaded into Dropbox.

Refresher training will be provided as follows:

- Video dissemination training: if a CSP scores below 70%
- Training on MIYCN and NSA: if a CSP scores below 75%
- PLA: If a CSP scores below 60%

On an ongoing basis, as need arises, refresher training on NSA and MIYCN will be provided for CSPs/CRPs/Supervisors who needs further help or newly recruited staff. These training programs will be conducted by the trainers who were trained by JSI.

2. Technical content training for AWWs and ASHAs (MIYCN)

After training of the specific CSPs and CRPs involved in the intervention, Anganwadi workers and ASHA workers from all UPAVAN villages (including control) will also be trained on maternal, infant and young child nutrition. The master trainers trained earlier by JSI will facilitate the training, but the JSI team will not be present. Training of the AWWs and ASHAs will start after the completion of the training of CSPs and CRPs and will continue from 15th May until end of October. There will be a total of 12 training programs in arm 1, 2 and 3

3. Video dissemination, home visit, and community mobilization training for CSPs

Three skills-based trainings will run during the second week of January 2017 for all CSPs involved in the UPAVAN intervention. Trainings will be the same for CSPs across all 3 intervention arms (A1, A2, A3). Video dissemination/ home visit/ community mobilization trainings will be conducted by the DG team and will follow the standard DG methodology. However, the UPAVAN team will provide inputs for video dissemination training (facilitation skills and home visits, in particular) specific to NSA and MIYCN. Updated formats for health and nutrition MIS will be integrated into this training as well.

Trainings will be conducted by Digital Green in collaboration with VARRAT and will be residential trainings.

Content of trainings includes but is not limited to:

- Video dissemination skills (pico projector handling, facilitation skills)
- MIS-related skills (attendance form and home visit form)
- Home visit skills (home visit form, reinforcement of messages, linking with AWW/ASHA workers)
- Community mobilization skills (introductory/launch event)

See DG website training page for video dissemination and video production related training materials

4. PLA training for CSPs involved in A3 of the UPAVAN project

CSPs and CRPs assigned to A3 will complete an additional training for conducting the PLA meetings.

The training will be delivered in five phases. The first phase of PLA training is planned from **1st to 3rd March, for 3 days of residential training** (after CSPs have been trained on NSA and MIYCN). Ekjut, with support from VARRAT and DG, will conduct the trainings; VARRAT will be managing training logistics.

The following people will be involved in PLA training:

- 23 CSPs,
- 2 CRPs (those for PLA-generated videos),
- A3 Supervisors and PLA supervisors
- A3 arm coordinator
- Program coordinator

All PLA trainings are planned to be residential to give ample time for practice and to encourage maximum participation.

The trainings will cover:

1. Introduction to PLA approach and the PLA cycle,
2. Facilitation skills, communication skills, leadership skills, conflict resolution via role play, meeting methods, picture cards, methods to address inequities,
3. Orienting to purposively seek participation of pregnant women and mothers of children under two, and ensuring that the marginalised people living in hamlets and remote villages do not get left out
4. Technical inputs on making stories, choosing feasible strategies, organizing meetings with the wider community.
5. Participatory exercises to facilitate discussion and action on videos, e.g. using demonstration of preparation of locally available foods, food ranking exercises, participatory food mapping, handwashing demonstrations.
6. Process and MIS data (group attendance, postponements, PLA meeting plan) formats, filling up meeting-specific forms and on participatory evaluation methods (how to get participants' feedback and report in the MIS forms).
7. How to engage frontline workers in the initial community meetings and throughout the PLA process.

5 Phases of PLA Training:

- 1st Training for PLA phase I = 3 days – will cover 4 PLA meetings + 1 meeting on the process of disseminating a video
- 2nd Training for Phase II of PLA Meetings = 3 days = 4 PLA Meetings + 1 Community Meeting + 1 meeting on the process of disseminating a video
- 3rd training for PHASE III of PLA Meetings = 3 days = 3 Video dissemination + 2 PLA Meetings
- 4th training for PHASE III of PLA Meetings = 3 days = 4 videos + 4 PLA Meetings
- 5th training for PHASE III & IV of PLA Meetings = 3 days = 2 videos + 4 PLA Meetings + 1 Community Meeting

Location = VARRAT office or place of convenience within Keonjhar district

Evaluation methods for both trainees and trainers during PLA training include the following:

1. Post-training, question and answer session to assess participants' knowledge retention of the contents and level of understanding as far as processes are concerned. *This will be an oral test and each participant will answer the question written on the chit which he/she picks up from a bowl*
2. Post-training written test, where all the participants will go through the same set of questions. *This will help us identify those CSPs who should be given more focus, by the supervisors subsequently (both in review meetings and meetings).*
3. The participants will also provide feedback and assess the methods and contents of the training and their level of understanding.

5. Data entry operator training

Data entry operators from VARRAT must be trained in how to enter the attendance and home visit form contents into COCO to ensure quality monitoring data collection. Digital Green will conduct a half day training for data entry operators on the fields on the form, how to enter into COCO, the frequency of data entry required, and what to do when there is missing data. Training will occur during the week of 15-19 May 2017 at VARRAT campus.

Harris-Fry H, *et al. BMJ Global Health* 2020; 5:e002384. doi: 10.1136/bmjgh-2020-002384

The following are guiding points for the review meeting documentation:

- Number of 1000-day women who are not attending and reasons why (reviewed every other month)
- New content that the community expressed an interest in
- Reasons any meeting or video dissemination didn't take place or was cancelled
- Problems with holding meetings or mobilizing beneficiaries
- Issues with finding space, wall, video and PICO functionalities
- Video content - feedback (positive or negative) on feasibility of practices being suggested—serious objections, what they liked/worked well
- Level of engagement of CSPs during meetings
- Home visits—not enough time, difficulty finding beneficiaries; time taken per visit on average and proportion of total workload
- PLA-meetings (A3 only)—meeting manual content, facilitation techniques that work well
- Community level response—FLWs coming, response from other community members
- Workload of CSPS—major complaints/issues
- Forms—any issues or clarifications required

e. HR capacity and post-training tests

Staff performance will be monitored on an ongoing basis using VARRAT's institutional line management mechanisms and review meetings.

POST-TRAINING

Post-training tests for trainings on NSA and MIYCN, use of pico projectors, and PLA facilitation will be conducted and documented per CSP. Refresher training will be provided to CSPs that score lower than the following thresholds:

- Video dissemination training: if a CSP scores below 70%
- Training on MIYCN and NSA: if a CSP scores below 75%
- PLA: If a CSP scores below 80%

Post-training test formats and results can be found in the UPAVAN Dropbox:

UPAVAN> Trainings > Post-Training Assessments

CSP CAPACITY BUILDING

CSPs are given a letter grade (A, B, or C) as part of the mediation observation format. CSPs scoring a 'B' grade or below are provided with a capacity building and support to ensure their further improvement. The arm coordinator/project coordinator who grades the CSP a B or C is responsible for documenting and implementing the CSPs' capacity building plans. These plans should be recorded regularly on the "CSP–QA Progress report" excel found in in the UPAVAN drobox:

UPAVAN > Capacity Building Plans > CSP- QA Progress report

This excel sheet is arm-wise and includes the following parameters: Observer name; CSP name; dissemination observation details including date of observation, video observed, overall dissemination observation score; parameters where the CSP scored 0 or 1; follow-up actions taken; dissemination observation follow-up date; and current score.

VARRAT also has a register to maintain staff profiles and monitor staff turnover.

Harris-Fry H, *et al. BMJ Global Health* 2020; 5:e002384. doi: 10.1136/bmjgh-2020-002384
